# Single-cell RNA sequencing reveals a landscape and targeted treatment of ferroptosis in retinal ischemia/reperfusion injury

**DOI:** 10.1186/s12974-022-02621-9

**Published:** 2022-10-26

**Authors:** Yangyang Li, Yuwen Wen, Xiuxing Liu, Zhuang Li, Bingying Lin, Caibin Deng, Ziyu Yu, Yingting Zhu, Ling Zhao, Wenru Su, Yehong Zhuo

**Affiliations:** State Key Laboratory of Ophthalmology, Zhongshan Ophthalmic Center, Sun Yat-Sen University, Guangdong Provincial Key Laboratory of Ophthalmology and Visual Science, Guangzhou, 510060 China

**Keywords:** Single-cell RNA sequencing, Retinal ischemia–reperfusion injury, Ferroptosis, Fer-1

## Abstract

**Background:**

The aim of this study was to establish a complete retinal cell atlas of ischemia–reperfusion injury by single-cell RNA sequencing, and to explore the underlying mechanism of retinal ischemia–reperfusion injury in mice.

**Methods:**

Single-cell RNA sequencing was used to evaluate changes in the mouse retinal ischemia reperfusion model. In vivo and in vitro experiments were performed to verify the protective effect of inhibiting ferroptosis in retinal ischemia–reperfusion injury.

**Results:**

After ischemia–reperfusion injury, retinal cells were significantly reduced, accompanied by the activation of myeloid and a large amount of blood-derived immune cell infiltration. The IFNG, MAPK and NFKB signaling pathways in retinal neuronal cells, together with the TNF signaling pathway in myeloid give rise to a strong inflammatory response in the I/R state. Besides, the expression of genes implicating iron metabolism, oxidative stress and multiple programed cell death pathways have changed in cell subtypes described above. Especially the ferroptosis-related genes and blocking this process could apparently alleviate the inflammatory immune responses and enhance retinal ganglion cells survival.

**Conclusions:**

We established a comprehensive landscape of mouse retinal ischemia–reperfusion injury at the single-cell level, revealing the important role of ferroptosis during this injury, and targeted inhibition of ferroptosis can effectively protect retinal structure and function.

**Supplementary Information:**

The online version contains supplementary material available at 10.1186/s12974-022-02621-9.

## Background

Retinal ischemia–reperfusion (I/R), a common cause of irreversible visual impairment, is involved in the pathological mechanisms of many eye diseases, including glaucoma, diabetic retinopathy, and retinal occlusion [1–3]. Retinal I/R is defined as an initial restricted retinal blood supply followed by perfusion restoration [3, 4]. This process results in retinal ganglion cells (RGC) death, morphological degeneration, function loss, and ultimately vision loss [5] due to free radical production, mitochondrial dysfunction, exacerbated oxidative stress, inflammatory response, and activation of calpains and caspases. Current treatments for retinal I/R injury include intraocular injections, eye drops, and surgery [6]. However, due to the complexity of the underlying mechanisms, no thoroughly effective treatments are available. The limitations of the available treatments motivated studies searching for alternatives with wide safety margins. Understanding the mechanisms controlling human retinal development is particularly important when treating vision-threatening diseases.

Previous studies have demonstrated that neuroinflammation was involved in I/R injury [7, 8]. The inflammatory response includes rapid and transient granulocyte infiltration, slow monocytes/macrophages accumulation, and resident microglia activation, proliferation, and mobilization to the vascular barriers [9]. Simultaneously, ischemia mediates retinal tissue damage and RGC death by triggering damage-associated molecular pattern-induced toll-like receptor 4 (TLR4), inflammasome-dependent neuroinflammation and microglial activation [7, 8]. Furthermore, cell deaths, including apoptosis, pyroptosis, and autophagy, has been implicated in inflammatory cytokine release [10, 11]. However, these studies focused on a limited number of cell types or mechanisms, lacking knowledge of the complex hierarchies and where the implicated genes are expressed. The full impact of I/R injury on the retina and the alternations in cell-type proportions have not been fully explored or understood. In addition, the unique interactions among the retinal cells under I/R conditions required further investigation. Therefore, a comprehensive retinal cell atlas that encompasses the influences of I/R at a single-cell resolution was urgently needed to integrate various retinal I/R interconnected components, pathways, and cell types.

Single-cell RNA sequencing (scRNA-seq) is a powerful and unbiased approach to comprehensively classify cell types and states based on their individual transcriptomes in health and disease. Its application has greatly boosted the understanding of the pathogenesis of many diseases [12–14]. Herein, we applied scRNA-seq to comprehensively characterize and compared mouse retinas of an I/R model and a wild-type (blank) control, aiming to identify marked changes in the transcriptome of various cell types. Ferroptosis occurred in most retinal cell types in response to I/R, particularly in the photoreceptors, glial cells, and RGCs. We found that ferrostatin-1 (Fer-1, a ferroptosis inhibitor) administration ameliorated RGC death, reduced the immune response induced by microglia, and protected retinal structure and function. These findings offer unique insights into potential strategies for treating diseases caused by retinal I/R injury, including glaucoma.

## Materials and methods

### Preparation of single-cell suspension of mouse retina

3 days after ischemia–reperfusion injury, 3 retinas were taken from the model group or the blank group, respectively, as a mixed sample, and the retinas were removed after perfusion with pre-cooled 0.9% normal saline. 2 mg/ml collagenase-D and 200 U/ml DNAseI were added for digestion, and an equal volume of 10% BSA was used to terminate the digestion, then cell suspension was collected. All cells were washed twice with PBS, and 10 μl samples were stained with Trypan blue for cell count. The number and viability of cells were calculated accurately. Adjust cell terminal concentration to about 1.0 × 10^6^ cell/ml.

### ScRNA-seq data alignment, processing, and sample aggregation

Gel Bead and Multiplex Kit, Chip Kit (10X Genomics) and Chromium Single Cell 5′ Library (10X Genomics, Genomics chromium platform Illumina NovaSeq 6000) were used to convert the harvested single-cell suspensions into barcoded scRNA-seq libraries. Single-cell RNA libraries was made up of the Chromium Single Cell 5′ v2 Reagent Kit (120237; 10X Genomics) according to the manufacturer’s instructions, and FastQC software was used to inspect library quality. All sequenced data were preliminarily processed by CellRanger software (version 4.0; 10X Genomics). The count pipeline of the CellRanger Software Suite was applied to demultiplexed and barcoded sequences. Seurat package (version 3.0) was applied to filtration, normalization, dimensionality reduction, cell clustering, and differential gene expression analysis of the processed data, on the foundation of the calculated single-cell expression matrix by CellRanger. Cells with less than 200 genes detected and a mitochondrial gene ratio of more than 20% were excluded. A total of 53,701 cells (Blank, 15,617 cells; Model, 38,084 cells) were analyzed after quality control.

### Dimensionality reduction and clustering analysis

The ‘‘NormalizeData’’ function was used to log-normalize the counts of each cell (1+ counts per 10,000). Dimensionality reduction was achieved by “RunPCA” function. Cells were visualized by means of a two-dimensional t-SNE algorithm in the ‘‘RunTSNE’’ function. The “FindNeighbors’’ and “FindClusters” functions were used to identify significant clusters at an appropriate resolution. The function “FindAllMarkers” served as identifying marker genes of each significant cluster. The top 10 most variable genes were extracted by the “FindVariableGenes” function in Seurat with default parameters.

### Differential expression analysis

Before running differential expression analysis, cell types which were missing or possessed no more than three cells in two groups were filtered out. To identify certain cell type between different groups, differential expression analysis was performed by the Wilcoxon rank-sum test implemented in the ‘‘FindMarkers’’ function of the Seurat package (version 3.0). A ferroptosis-related DEG data set was established (*P* value < 0.05, |Log_2_FC| > 0.25) after identification of DEG between groups.

### Gene functional annotation

As a web-based portal, Metascape (www.metascape.org) was used to conduct GO and pathway analysis with the input of DEG [15]. The top 10 of 30 GO biological processes and pathways among groups and clusters were visualized by ggplot2 R package.

### Determination of cell–cell interactions

Cell-level interactions among different cell types was analyzed under the help of processed scRNA-seq data. CellChat (https://github.com/sqjin/CellChat), an R package could quantitatively calculate the intercellular communication networks and predict the main signal pathways, was used to implement the signaling pathway networks visualization. Only receptors and ligands expressed in at least 10% of specific cells were used for further analyzed, besides, communication was considered not theoretically existed if the ligand and receptor were not detected. TBtools (www.tbtools.com) was applied to data normalization and heatmap drawing.

### Scoring of biological processes

Individual cells were scored by gene signatures representing certain biological functions, then calculated with the average of normalized expression of corresponding genes. Full gene lists with biological functional signature were obtained in GO and KEGG database. For instance, the inflammatory response score was determined by calculating the average expression of genes in the GO term “inflammatory response” (GO: 0006954). Ferroptosis-related gene signatures were obtained from the KEGG Pathway data set “Ferroptosis” (ko04216).

### I/R model and ferrastatin-1 treatment

Six-week-old C57BL/6J male mice were purchased from the Guangdong Medical Laboratory Animal Center. All animals were kept in a specific pathogen-free facility in Animal Laboratories of Zhongshan Ophthalmic Center and the experiments were approved by the Institutional Animal Care and Use Committee of Zhongshan Ophthalmic Center, Sun Yat-sen University. The retinal I/R model were established as previously described [16]. In brief, a 30-gauge needle containing a balanced salt solution was cannulated into the anterior chamber to maintain the IOP at 70 mmHg. The sham operation, which acted as the control, was implemented a same operation without elevating the IOP. After 60 min, the IOP was normalized by carefully withdrawing the needle. Tobramycin ointment (Alcon, USA) was used to prevent a bacterial infection. It's worth noting that 30 μg/ml ferrostatin-1 was injected intravitreally to the operated eye immediately after I/R injury.

### Histological examination

7 days after the operation, heart perfusion was applied to euthanized mice using normal saline and 4% paraformaldehyde (PFA). Whole eye balls were fixed in 4% PFA at 4 °C over night and followed by paraffin fixation. Every sample was sectioned into 10-μm-thick slices through the optic nerve plane and followed by H&E staining. Four cross-sectional measurements around the optic nerve (within 1 mm) for every retina were chosen to quantify the inner plexiform layer (IPL) thickness using a Leica DM6B device and analyzed by ImageJ software (https://imagej.nih.gov/ij/).

### Immunofluorescence staining and examination

Retinas were collected carefully then fixed by 4% paraformaldehyde, permeabilized by 0.3% Triton X-100, and blocked by 5% bovine serum albumin. For whole-mount staining, collected samples were incubated with primary antibodies at 4 °C overnight, followed by a species-compatible secondary antibody for 2 h at room temperature. The sources and dilutions of antibodies listed as Additional file 2: Table S1. Cell nuclei were stained by 1 ng/ml 40,6-diamidino-2-phenylindole (DAPI; Beyotime, China) for 10 min. Images were captured by a Zeiss LSM 880 confocal laser-scanning microscope and processed by ZEN 2 and Adobe Photoshop CS6.

### Cell culture of primary retinal microglia

Retinal microglia were isolated from the retina of 3–5 day C57BL/6J neonatal wild-type mice as previously described with a slight modification [17]. In brief, mixed glial cultures were separate from the retinas, followed by a chemical dissociation (trypsin–EDTA 0.05%, Gibco) conducted at 37 °C with a gentle blow for 1 min until the tissue masses disappeared and digestion was terminated with the equivalent amount of DMEM/F12 (Gibco, USA) supplemented with 10% (vol/vol) FBS. Retinal mix cells were then resuspended in DMEM/F12 (Gibco, USA) supplemented with 10% (vol/vol) FBS, and continued to culture until reaching confluency. Detached microglia were collected by a horizontal shaker at 180 rpm, 37 °C for 2 h. Transferred the collected cells into a 6-well plate and continued to culture for another 1–2 weeks before an identification by flow cytometry.

### Establishment of the OGD/R model

As an in vitro model of retinal ischemia/reperfusion, we used oxygen–glucose deprivation/reperfusion (OGD/R) in primary microglia cells. In brief, cells were cultured in glucose-free DMEM(Gibco) after washing twice by PBS(Gibco), then subjected to an anoxic chamber (Billups-Rothenberg, Inc., USA) containing 5% (vol/vol) CO2 and 95% (vol/vol) N2 at 37 °C for 3 h. For the same duration, cells incubated in serum-free medium supplemented with 4.5 g/l d-glucose under normoxic conditions acted as control. At the end of the exposure period, cells were then reoxygenated [5% (vol/vol) CO_2_ and 95% (vol/vol) air] with normal medium for 24 h.

### Western blotting

Total protein was isolated from the retinas with radioimmunoprecipitation assay (RIPA) lysis solution (Beyotime, China) and run on 12% (wt/vol) gradient polyacrylamide gels following a standard protocol. The expression of target proteins was normalized to β-actin obtained from the same sample (taken as 1.0) and then quantified using ImageJ Software (https://imagej.nih.gov/ij/). The primary antibodies and dilutions were listed in Additional file 2: Table S1.

### Quantitative real-time PCR and qPCR

Total RNA was extracted from the retina and cultured cells with TRIzol Reagent (Invitrogen) according to the manufacturer’s instructions. cDNA was synthesized with the PrimeScript RT Master Mix (TaKaRa) according to a standard protocol. Quantitative analysis was conducted by the Light Cycler 480 Real-Time PCR System (Roche Molecular Systems, Inc., SUR). The expression of target mRNAs was measured and normalized to that of GAPDH. Additional file 2: Table S2 lists the primers.

### Flow cytometry and detection of reactive oxygen species (ROS)

Cells were isolated from eye balls and prepared to single-cell suspensions. The cell activity was detected with Zombie NIR™ Fixable Viability Kit (APC-Cy7, catalog 423105, Biolegend, San Diego, CA, USA). Then, cells were stained with fluorochrome-conjugated mAbs surface markers CD11b (Bv605, catalog 101237, Biolegend) for 15 min. For detection of reactive oxygen species (ROS), cells next were incubated with H2DCFDA (FITC, catalog HY-D0940, MedChemExpress, Monmouth Junction, NJ, USA) to detect the ROS at 37 °C for 20–30 min. Finally, washing cells with cold PBS for three times before analyzing with flow cytometry (BD LSR Fortessa, BD Bioscience, San Jose, CA, USA). All data were analyzed using FlowJo (TreeStar, Ashland, OR, USA).

### Statistics

Data quantification were performed blindly and presented as mean ± SE of measurement (SEM). Data were analyzed statistically using one-way ANOVA with Bonferroni’s post hoc test for comparisons of three and more groups or two-tailed Student’s t test for two group comparisons. To assess significance, a value of *P* < 0.05 was considered statistically significant (*P < 0.05; **P < 0.01; ***P < 0.001; ****P < 0.0001). The sample sizes and P values are indicated in the figure legends.

### Data availability

The scRNA-seq data are deposited in the Genome Sequence Archive in BIG Data Center, Beijing Institute of Genomics (BIG, https://bigd.big.ac.cn/gsa/), Chinese Academy of Sciences, under the Project Accession No. PRJCA008174 and GSA Accession No. CRA006042.

## Results

### ScRNA-seq yielded retinal cell profiling in mice with I/R injury

Single-cell transcriptome analysis was performed on retinal tissue of 8-week-old blank and retinal I/R mice to generate a comprehensive cell atlas of this I/R model. We then used 10× Genomics to generate a barcoded scRNA-seq library of the cell suspension samples. The sequencing data were processed using Cell Ranger software (version 3.1.0) to generate a matrix of unique molecular identifiers that was analyzed using Seurat R Package v3 [18]. scRNA-seq profiles passing quality control were corrected for technical 10× run batch effects using Harmony [19]. Unsupervised clustering of the Harmony-corrected data, followed by two-dimensional t-distributed stochastic neighbor embedding (tSNE), revealed 12 distinct molecular clusters, consistently clustering the various cell types into distinct regions.

**Fig. 1:**
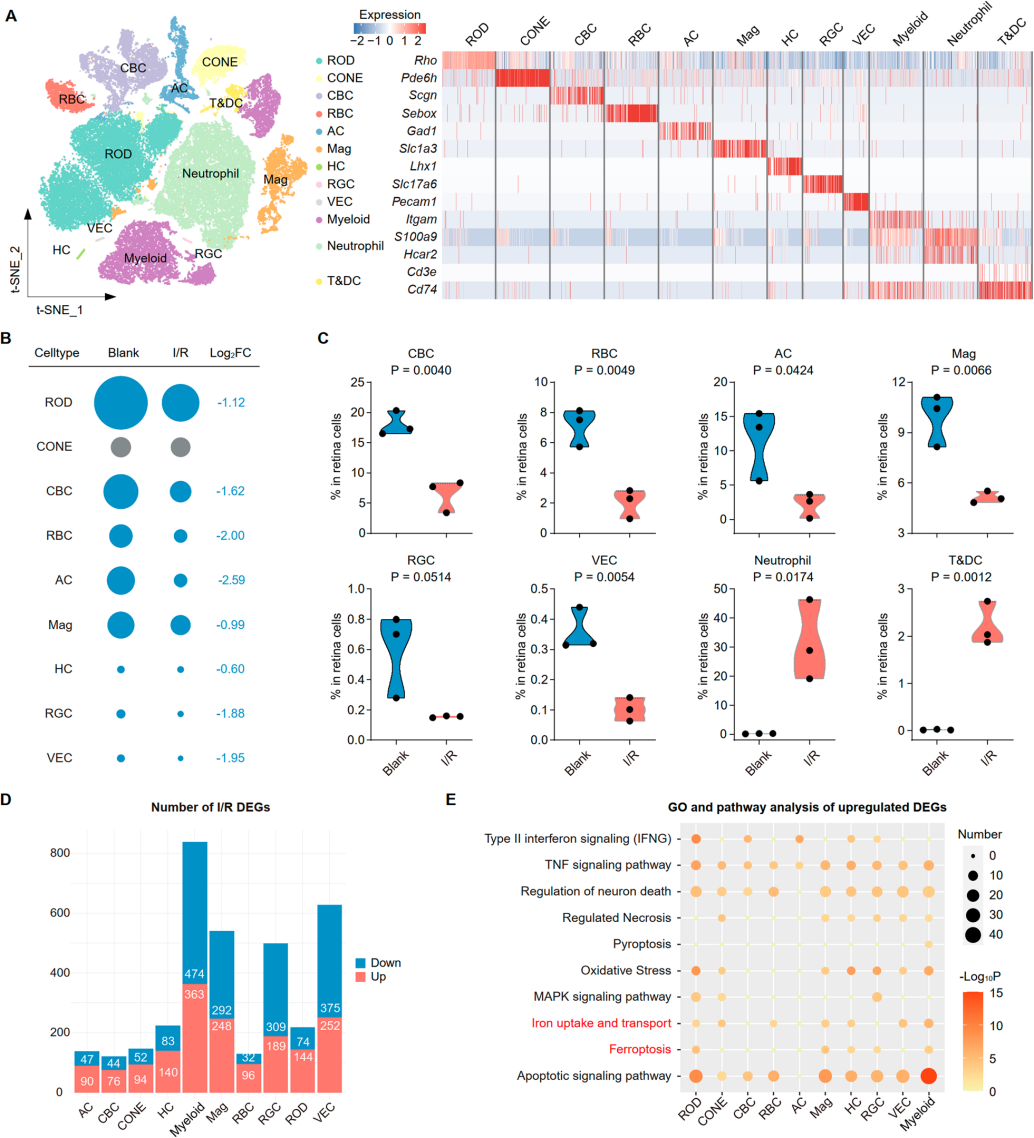
scRNA-seq reveals altered retinal heterogeneity in mice with I/R injury. **A** Clustering strategy of major retinal cell populations identifying 12 cell types (left) based on the scaled expression heatmap of canonical markers for each cluster (right). Color scheme is based on *z*-score distribution from − 2 (blue) to 2 (red). **B** Relative changes in cell ratios in different cluster between Blank and I/R groups. The numbers on the right indicate the Log_2_FC values of the cell ratios. **C** Violin plots showing the proportion of 8 clusters in retinal cells between Blank and I/R groups (*n* = 3/group). *P* value were calculated using a Wilcoxon rank-sum test. **D** t-SNE distribution showing I/R-upregulated DEGs numbers from 12 clusters. **E** GO and pathway enrichment analysis of I/R-upregulated DEGs in 12 clusters of the I/R model. *P* value was derived by a hypergeometric test

We analyzed the distribution of the four cell lineages in the retina, including seven neuronal classes, Rod, Cone, cone bipolar cell (CBC), rod bipolar cell (RBC), amacrine cell (AC), horizontal cell (HC), and retinal ganglion cell (RGC), one glial class, macroglia (Mag), three immune classes, monocyte macrophage and microglia (Myeloid), Neutrophil, and T cell and dendritic cells (T&DC), and vascular endothelial cell (VEC), based on canonical markers and the most variable upregulated genes (Fig. 1A). Notably, we found that a group of Myeloid cells with high expression of ITGAM appeared during retinal I/R injury, including retina microglia, blood-derived macrophages and monocytes (Additional file 1: Fig. S1A). The number of cells and relative per-class cell types in the retinal I/R and blank groups are depicted in Additional file 1: Fig. S1B. Many clusters were differentially represented in the two groups. Myeloid, and T&DC subsets were over-represented in the I/R model and ROD, CBC, RBC, AC, RGC, and VEC subsets were underrepresented (Fig. 1B). Compared to the blank group, the I/R model had a markedly higher proportion of retinal cells in the Neutrophil and T&DC subsets and lower in the CBC, RBC, AC, Mag, RGC, and VEC subsets (*p* < 0.05; Fig. 1C). Four subsets (CONE, ROD, HC, and Myeloid) were similarly present in both groups (Additional file 1: Fig. S1B). scRNA-seq analysis revealed cell cluster differences between the groups.

On condition of the exclusion of subsets of cells with a percentage of less than 0.1%, we conducted differentially expressed gene (DEG) analysis comparing the I/R and blank groups to further understand the gene changes in the retinal I/R model. tSNE is the number of upregulated DEG. Of these, over 400 were primarily expressed in the RGC and Myeloid subsets (Fig. 1D). Using the Kyoto Encyclopedia of Genes and Genomes (KEGG) database, we showed that these upregulated genes were mainly associated with cell death regulation (e.g., apoptotic signaling pathway, ferroptosis, pyroptosis, necrosis, and neuronal death), oxidative stress, iron intake and transport, type II interferon signaling (IFNG), and the TNF and MAPK signaling pathways (Fig. 1E).

Taken together, we constructed an integrative transcriptional atlas containing multiple neuron and immune cell subpopulations and established a cellular profile to further understand the dynamic changes in immune cells and neurons during I/R.

### Classification and differential expression gene (DEG) analysis of the retinal photoreceptor cells

**Fig. 2:**
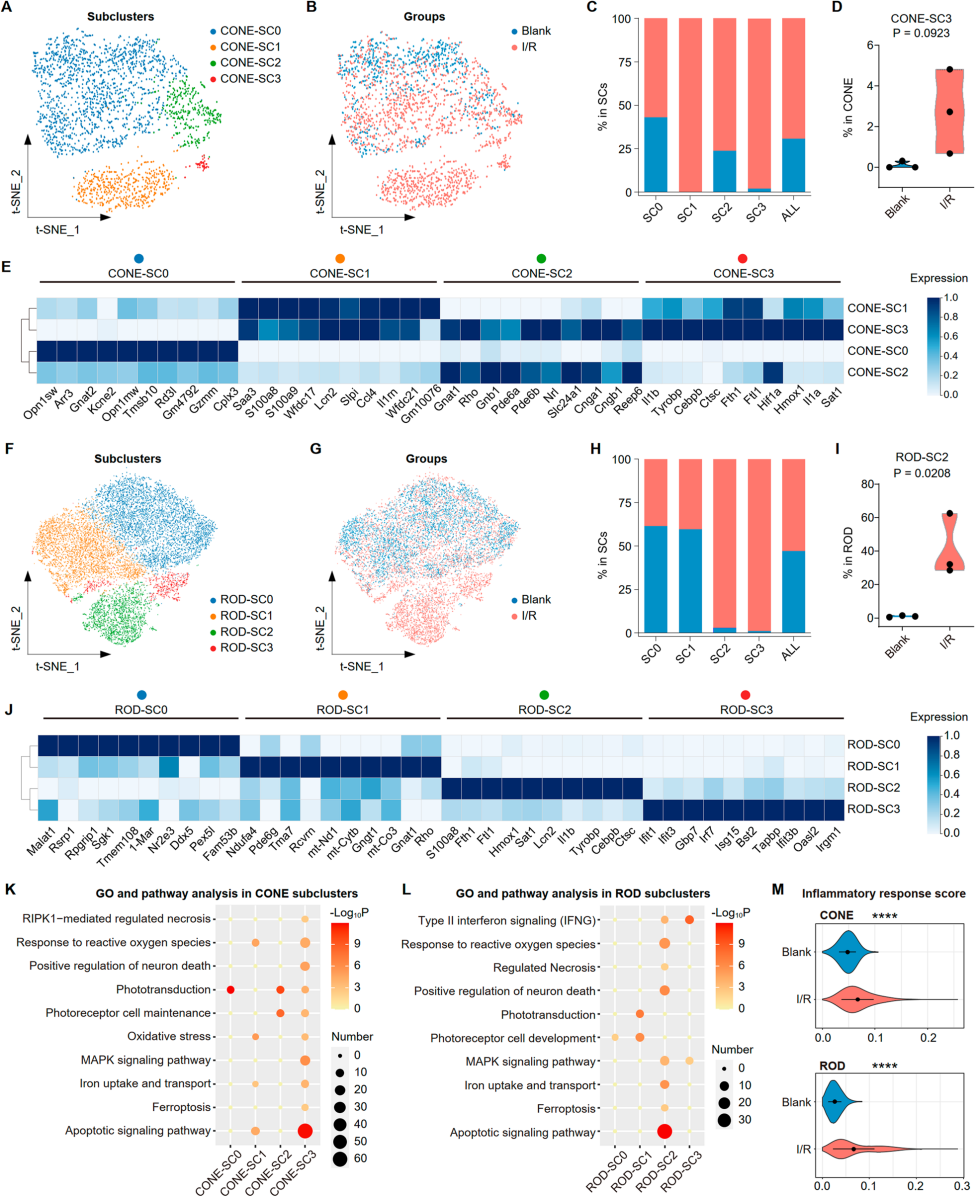
I/R injury induced an expansion of photoreceptor subclusters with ferroptosis. **A** t-SNE distribution showing 4 subclusters in CONE. **B** t-SNE distribution showing groups (Blank and I/R) in CONE. **C** Bar plots showing cell abundances across CONE-SCs (*n* = 4) for the Blank and I/R groups. **D** Violin plots showing the proportion of CONE-SC3 in CONE between Blank and I/R groups (*n* = 3/group). *P* value were calculated using a Wilcoxon rank-sum test. **E** Heat map representing the scaled expression values of the top 10 genes defining each CONE-SCs. **F** t-SNE distribution showing 4 subclusters in ROD. **G** t-SNE distribution showing groups (Blank and I/R) in ROD. **H** Bar plots showing cell abundances across ROD-SCs (*n* = 4) for the Blank and I/R groups. **I** Violin plots showing the proportion of ROD-SC2 in ROD between Blank and I/R groups (*n* = 3/group). *P* value were calculated using a Wilcoxon rank-sum test. **J** Heat map representing the scaled expression values of the top 10 genes defining each ROD-SCs. **K** GO and pathway enrichment analysis of the CONE-SCs clustering DEGs. P value was derived by a hypergeometric test. **L** GO and pathway enrichment analysis of the ROD-SCs clustering DEGs. *P* value was derived by a hypergeometric test. **M** Violin plots showing the inflammatory response score between Blank and I/R groups among CONE (up) and ROD (down) clusters

We examined I/R-induced alterations in photoreceptor cells, because they are highly sensitive to I/R and lost shortly after it is induced [20]. We identified two clear photoreceptor clusters, the rods that selectively express rhodopsin and cones that express short- and medium-wavelength opsins (Additional file 1: Fig. S1D). Cone photoreceptors in both groups comprised four subclusters (SCs), SC0 to SC3 (Fig. 2A, B). Although the groups were similar in cones, they differed in their cone SC proportions (Fig. 2C). We observed that the proportion of SC0, which showed high expression of Opn1sw, Arr3, Opn1mw, and other cone signature genes, decreased in the I/R group (Fig. 2C, E). Notably, SC1 and SC3 were found almost exclusively in the I/R group (Fig. 2C). Higher Neutrophil-derived protein S100a8/a9 expression was confirmed in SC1, whereas SC3 was enriched for pro-inflammatory cytokine genes (Ilβ, Il1a) and ferroptosis-related genes (Fth1, Flt1, and Hmox1; Fig. 2D, E). Similarly, the rods were similar in the two groups and showed four distinct subclusters (Fig. 2F, G, Additional file 1: Fig. S1E). The percentage of SC2 and SC3 increased dramatically after I/R injury, particularly SC2 that was enriched in pro-inflammatory (S100a8 and Il1b) and iron intake and transport (Fth1, Flt1, and Hmox1) genes (Fig. 2H–J).

We next explored the biological implications of the upregulated DEG through gene ontology (GO) and pathway analyses for each SC in the cone and rod photoreceptors. The commonly upregulated genes in cone-SC3 and rod-SC2 after I/R were enriched in apoptotic signaling pathways, ferroptosis, iron intake and transport, and cellular responses to stress (Fig. 2K, L). While the inflammation-related GO and pathway enrichment analysis showed highly expressions of TNF signaling pathway, NF-kappa B signaling pathway, inflammatory response, IL-17 signaling pathway and cellular response to IL-1 in cone-SC3 and rod-SC2 (Additional file 1: Fig. S1G, H). Next, we calculated the inflammatory response score in CONE and ROD, respectively, finding that the values of the I/R group were higher than those of the blank group in both cell types (Fig. 2M).

Hence, I/R-induced damage to the photoreceptor cells could be attributed to a reactive increase in specific SCs related to inflammation and ferroptosis.

### Ferroptosis is involved in I/R-induced RGC damage

**Fig. 3:**
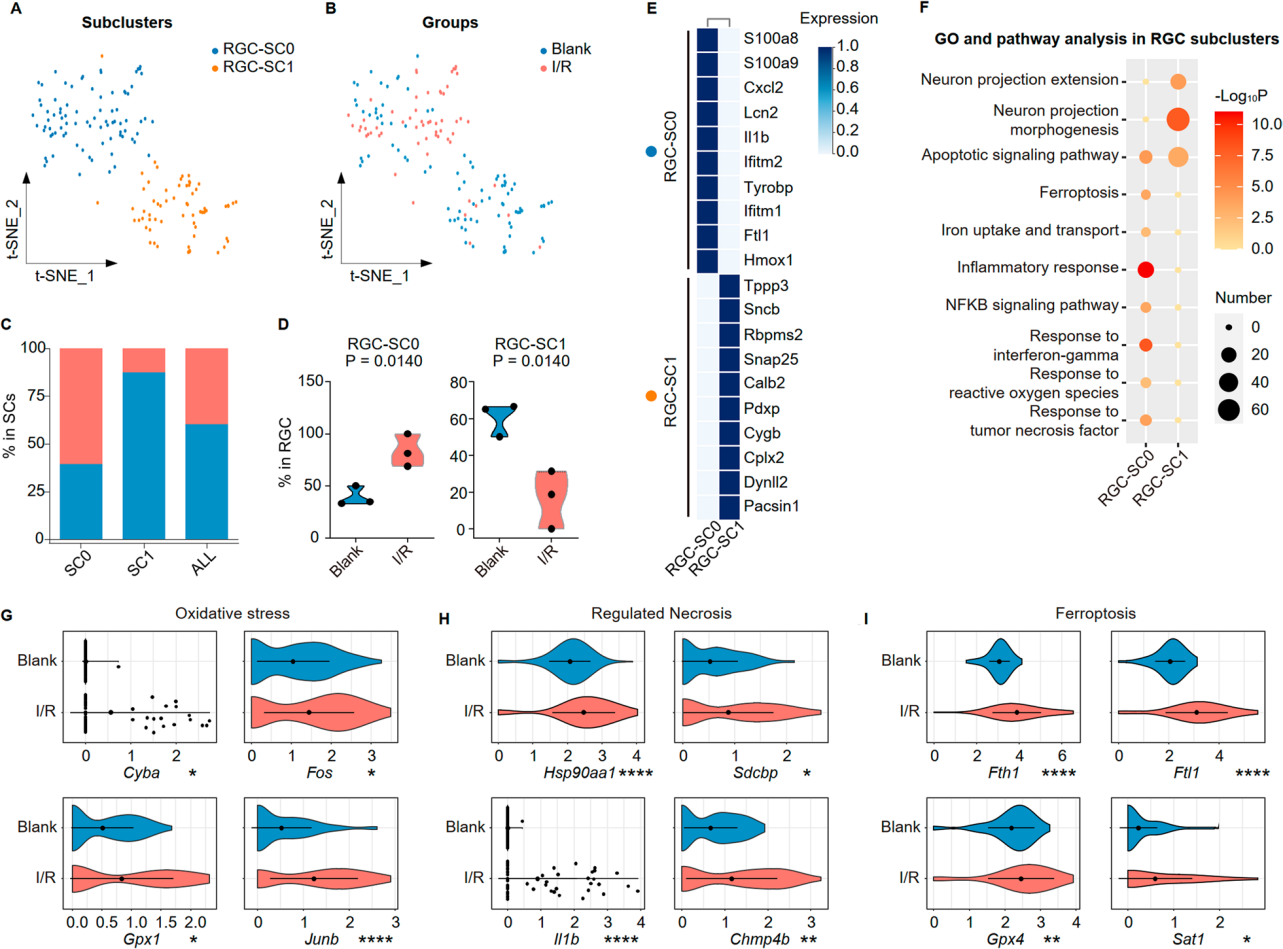
I/R injury induced an expansion of RGC subclusters with ferroptosis. **A** t-SNE distribution showing 2 subclusters in RGC. **B** t-SNE distribution showing groups (Blank and I/R) in RGC. **C** Bar plots showing cell abundances across RGC-SCs (*n* = 2) for the Blank and I/R groups. **D** Violin plots showing the proportion of RGC-SCs in RGC between Blank and I/R groups (*n* = 3/group). *P* value were calculated using a Wilcoxon rank-sum test. **E** Heat map representing the scaled expression values of the top 10 genes defining each RGC-SCs. **F** GO and pathway enrichment analysis of the RGC-SCs clustering DEGs. *P* value was derived by a hypergeometric test. **G** Violin plots showing the expression of genes related to oxidative stress between Blank and I/R groups. **H** Violin plots showing the expression of genes related to regulated necrosis between Blank and I/R groups. **I** Violin plots showing the expression of genes related to ferroptosis between Blank and I/R groups

We identified the RGC based on the expression of Slc17a6, which encodes the transporter Vglut2, and Rbpms, which encodes an RNA-binding protein with multiple splicing. These markers were weakly expressed in other AC and HC clusters. Our analysis divided the RGC into two subclusters based on their canonical markers (Fig. 3A). SC0 was highly represented in the I/R group and SC1 in the blank group (Fig. 3B–D). Cells in SC0 expressed high levels of S100a8, S100a9, Cxcl2, Il1b, Flt1, and Hmox1, genes related to ferroptosis, iron uptake and transport, and immune response. Cells in SC1 expressed high levels of Tppp3, Calb2, and Cygb, genes related to neuron projection morphogenesis and extension and the apoptotic signaling pathway (Fig. 3E, F).

Next, we adopted a violin-plotted feature genes of well-established forms and several key events of RGC damage, including oxidative stress (Cyba, Fos, Gpx1, and Junb), regulated necrosis (Hsp90aa1, Sdcbp, Il1b, and Chmp4p), and ferroptosis (Fig. 3G, H). Interestingly, we found that ferroptosis genes (Fth1, Flt1, Gpx4, and Sat1) were specifically enriched in RGC in the I/R retina (Fig. 3I), indicating that RGC primarily experienced ferroptosis during I/R. These findings demonstrated that I/R retinas were characterized by the emergence of ferroptosis-sensitive RGC-SC0.

### Retinal I/R led to ferroptosis-related changes in glial cells

Multiple glial cell types are activated during I/R. Macroglia, including astrocytes and Müller glia, undergo reactive gliosis after the retina and/or optic nerve is damaged, and interact with microglia to mediate the release of inflammatory cytokines. These cytokines were hypothesized detrimental or beneficial to the survival of RGC and their axon regeneration [20, 21]. We divided the macroglia into seven SCs (Additional file 1: Fig. S2A). The I/R mice had higher levels of SC1, SC2, SC3, and SC5, whereas the blank mice had larger proportions of SC0, SC4, and SC6 (Additional file 1: Fig. S2B–E). The known specific markers of Müller glia, Glul, Clu, and Dkk3, were upregulated in SC0. SC6 was classified as an astrocyte subcluster based on the expression of Gfap (Additional file 1: Fig. S2F). SC3 in the I/R group particularly stood out. Closer examination revealed significant enrichment in acute phase inflammation (Saa3, S100a8, S100a9, and Cxcl2) and ferroptosis (Fth1, Ftl1, and Hmox1) genes (Additional file 1: Fig. S2F). Furthermore, we found unique expression of some rod markers in SC1, including Rho and Gngt1, indicating that macroglia might play an important role in photoreceptor damage (Additional file 1: Fig. S2F, G).

GO analysis of DEG between the macroglia subclusters in I/R and blank retinas showed that SC3 was enriched in pathways, such as cell death (apoptosis signaling pathway, regulated necrosis, and ferroptosis), oxidative stress, iron uptake and transport, and immune response (MAPK pathway and response to interferon-gamma; Additional file 1: Fig. S2H). SC0, SC1, and SC4 were associated with neuronal system and neuron projection morphogenesis and extension (Additional file 1: Fig. S2H).

**Fig. 4:**
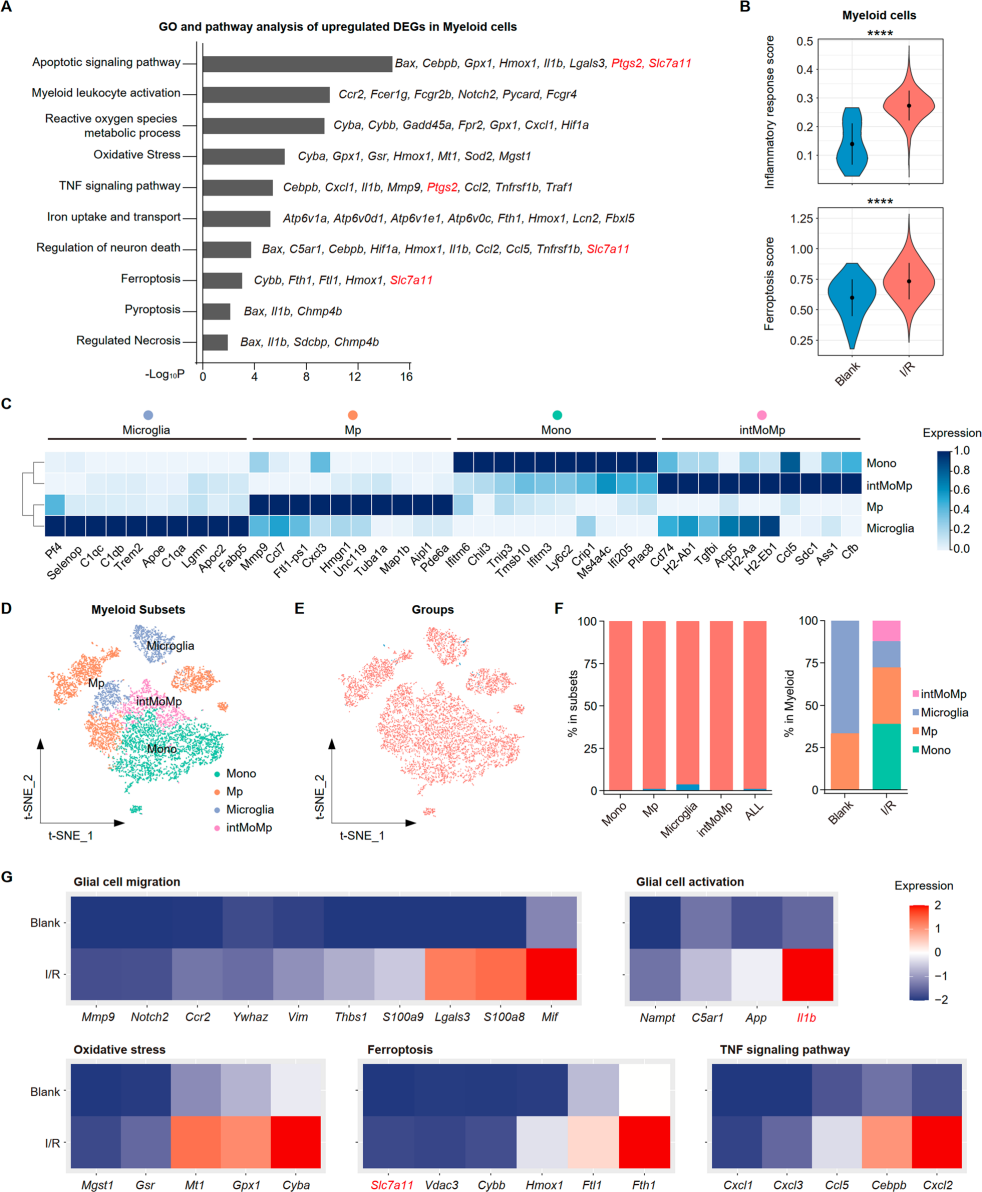
I/R injury induced an expansion of Myeloid subclusters with ferroptosis. **A** GO and pathway enrichment analysis of the Myeloid cells clustering DEGs. *P* value was derived by a hypergeometric test. **B** Violin plots showing the inflammatory response score (up) and ferroptosis score (down) between Blank and I/R groups. **C** Heat map representing the scaled expression values of the top 10 genes defining each Myeloid subsets. **D** t-SNE distribution showing 4 subclusters in Myeloid. **E** t-SNE distribution showing groups (Blank and I/R) in Myeloid. **F** Bar plots showing cell abundances across Myeloid (*n* = 4) for the Blank and I/R groups. **G** Heat maps demonstrating up-regulated genes and pathways in Myeloid during retina I/R injury

Myeloid activation is an early alteration during retinal I/R. Our previous study showed that the myeloid act as critical mediators, orchestrating neuroinflammation progression through pro-inflammatory cytokines [11]. Under stress, gliosis in retinal glia, such as macroglia, can be triggered by myeloid activation through increased cytokine levels [22]. Similarly, pathway-enrichment analysis with DEG obtained through unsupervised clustering analysis found apoptotic signaling pathways and the regulation of neuron death, driven in part by the upregulation of genes associated with ferroptosis, including Ptgs2 and Slc7a11. Genes involved in oxidative stress (e.g., Homx1 and Sod2) and iron uptake and transport (e.g., Atp6v1a, Atp6v0d1 and Fth1) were also upregulated (Fig. 4A and Additional file 1: Fig. S3A). The increased cell death pathways’ activities after I/R injury, particularly ferroptosis-related genes, demonstrated that I/R induced ferroptosis. We calculated the scores in Blank and I/R group to assess the extent of this phenomenon, finding that the latter exhibited higher inflammatory response score and ferroptosis score (Fig. 4B).

For further study, myeloid were subdivided into 4 subgroups. C1qa and C1qb positive microglia, Ly6c2 (high) and Tgfbi (low) monocytes (Mo), Ly6c2 (high) and Tgfbi (high) mononuclear macrophage intermediates(intMoMp), and the remaining macrophages (Mp) were isolated (Fig. 4C, D and Additional file 1: Fig. S3B). By comparing cell proportions, we found that Mo and intMoMp were newly emerged cell types after retina I/R injury (Fig. 4E, F).

We then focused on the up-regulated genes and pathways in myeloid during retina I/R injury, and found that genes related to glial migration and activation increasing apparently, as well as the oxidative stress, iron death, and TNF pathways (Fig. 4G). Further analysis also showed that retina I/R led to rises in the inflammatory response scores and ferroptosis scores in myeloid (Additional file 1: Fig. S3C).

In summary, the glial cells mediate inflammation and ferroptosis during I/R through multiple mechanisms and interactions.

### Aberrant cell–cell communication patterns may contribute to inflammatory tissue injury after inducing I/R

**Fig. 5:**
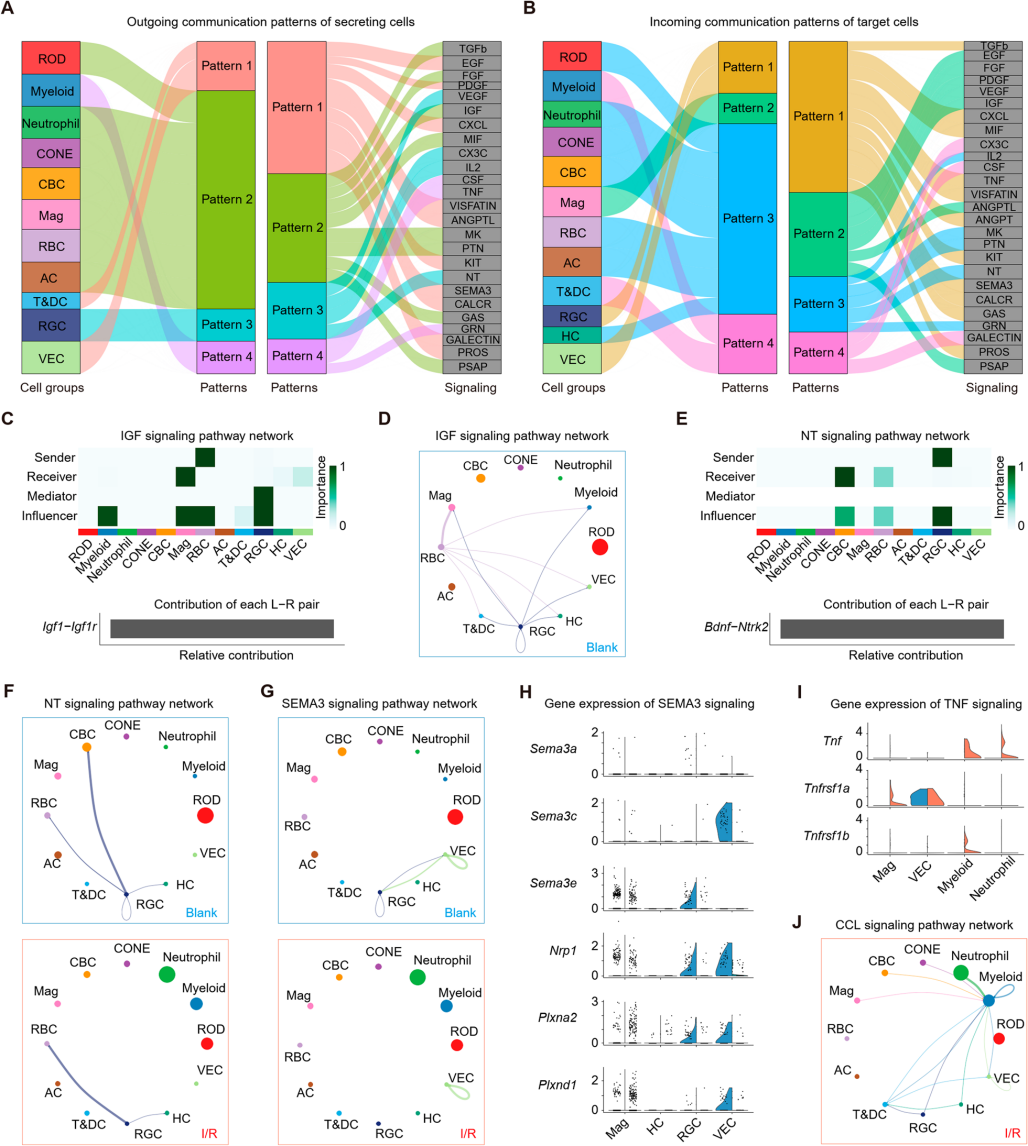
Analysis of cell–cell communication patterns differences between Blank and I/R. Sankey plots showing the inferred outgoing communication patterns of secreting cells (**A**) and incoming communication patterns of target cells (**B**), which shows the correspondence between the inferred latent patterns and cell groups, as well as signaling pathways. The thickness of the flow indicates the contribution of the cell group or signaling pathway to each latent pattern. **C** Heatmap shows the role and importance of each cluster in IGF signaling network (up). Bar plots showing the relative contribution of each ligand–receptor pair in IGF signaling pathway (down). **D** Circle plot showing the inferred IGF signaling networks in Blank group. **E** Heatmap shows the role and importance of each cluster in NT signaling network (up). Bar plots showing the relative contribution of each ligand–receptor pair in NT signaling pathway (down). **F** Circle plot showing the inferred NT signaling networks between Blank and I/R groups. **G** Circle plot showing the inferred SEMA3 signaling networks between Blank and I/R groups. **H** Violin plots showing the expression of genes related to SEMA3 signaling between Blank and I/R groups. **I** Violin plots showing the expression of genes related to TNF signaling between Blank and I/R groups. **J** Circle plot showing the inferred CCL signaling networks in I/R group

Cell-to-cell interactions orchestrate homeostasis and single-cell functions [23]. We explored intercellular signaling in I/R using CellChat, a tool that infers intercellular communication based on the expression of ligand–receptor pairs. Based on the pattern recognition method employed in CellChat, we first explored patterns shared by several cell populations to study how multiple cell groups and signaling pathways coordinated to function in normal retinal environment. The application of this analysis uncovered four outgoing (Fig. 5A) and four incoming (Fig. 5B) signaling patterns. A large portion of the outgoing retinal cell signaling was characterized by outgoing pattern #2 that represented multiple pathways, including TGFβ, FGF, IGF, and PSAP. Outgoing RGC signaling was characterized by outgoing pattern #3 that represented pathways, such as VEGF, IL2, and neurotrophin (NT; Fig. 5A). Target cell communication patterns (Fig. 5B) showed that incoming Mag signaling was dominated by incoming pattern #2 that included growth factor signaling (EGF, FGF, PDGF, VEGF, IGF), important for retinal lamination and photoreceptor development. Most incoming RGC signaling was characterized by incoming pattern #1, driven by the TGFb and Semaphorin 3 (SEMA3) pathways (Fig. 5B). These results showed that distinct retinal cell types (e.g., RGC and VEC) could rely on largely overlapping signaling networks. Certain cell types, such as glia and photoreceptor cells, simultaneously activated multiple growth signaling pathways, whereas immune cell types, such as T&DC and myeloid, relied on fewer signaling pathways.

Next, we explored the effects of I/R on intercellular interactions. Previous studies have shown that canonical IGF signaling activation was required for retinal lamination and photoreceptor development [24, 25]. Indeed, centrality analysis-specific signaling network identified RBC as the most prominent source of the Igf1 ligand (Fig. 5C), acting on Mag. IGF pathways were only active in the blank group (Fig. 5D, Additional file 1: Fig. S3D). NT signaling has multiple neuroprotective functions, including preventing retinal damage, with the Bdnf–Ntrk2 ligand–receptor pair being a major signaling driver (Fig. 5E). Our data revealed that NT signaling action on CBC was reduced in the I/R group, driven by Bdnf and Ntrk2 in RGC and CBC, respectively (Fig. 5F, Additional file 1: Fig. S3E). Next, we explored the signaling pathways activated by RGC. It was reported that the SEMA3 family was implicated in regulating developmental aspects of the visual system by affecting RGC maturation and guiding the RGC into the superior colliculus [26, 27]. VEC and RGC were the primary ligand sources in the SEMA3 signaling network, acting both in autocrine and paracrine manners (Fig. 5G, Additional file 1: Fig. S3F). Notably, we found that I/R reduced the expression of genes related to the SEMA3 signaling pathway, mainly between VEC and RGC (Fig. 5G, H). These results suggested that I/R might attenuate normal growth signaling among retinal cells, leading particularly to changes in the maturation and function of glial cells, CBC, and RGC, and the resulting retinal damage.

We also explored the influence of myeloid on intercellular interactions. The pattern recognition analysis found that myeloid dominantly drove CSF and TNF signaling, indicated by outgoing pattern #4 (Fig. 5A). CSF signaling is critical for myeloid cell proliferation [28], while TNF signaling is involved in cell death and inflammatory activation of myeloid [29]. Both could induce inflammatory tissue injury following I/R. Interestingly, the TNF signaling pathway output differed between the blank and I/R groups, with myeloid in the blank group and Neutrophil in the I/R group (Additional file 1: Fig. S3G). Tnf (a ligand) and Tnfrsf1a and Tnfrsf1b (its receptors) were increased in myeloid and Mag cells (Fig. 5I), suggesting that I/R induced TNF signaling pathway activation in glial cells. The CCL signaling pathway is important for myeloid activation and migration. This pathway was only present in the I/R group (Fig. 5J). Neutrophil and myeloid were the primary CCL sources acting on myeloid, with the Ccl3–Ccr1, Ccl3–Ccr5, and Ccl4–Ccr5 ligand–receptor pairs being the main signaling contributors (Additional file 1: Fig. S3H). We found that I/R increased the expression of Ccl3, Ccl4, Ccr1, and Ccr5, mainly in myeloid (Additional file 1: Fig. S3I).

Altogether, these findings revealed that specific intercellular interactions were affected by I/R. The reduced nerve growth and visual system development signaling and increased immune cell activation signaling might be the basis for inflammatory tissue injury after I/R.

### Ferrostatin-1 directly reduced retinal ferroptosis and enhanced RGC survival after I/R

**Fig. 6:**
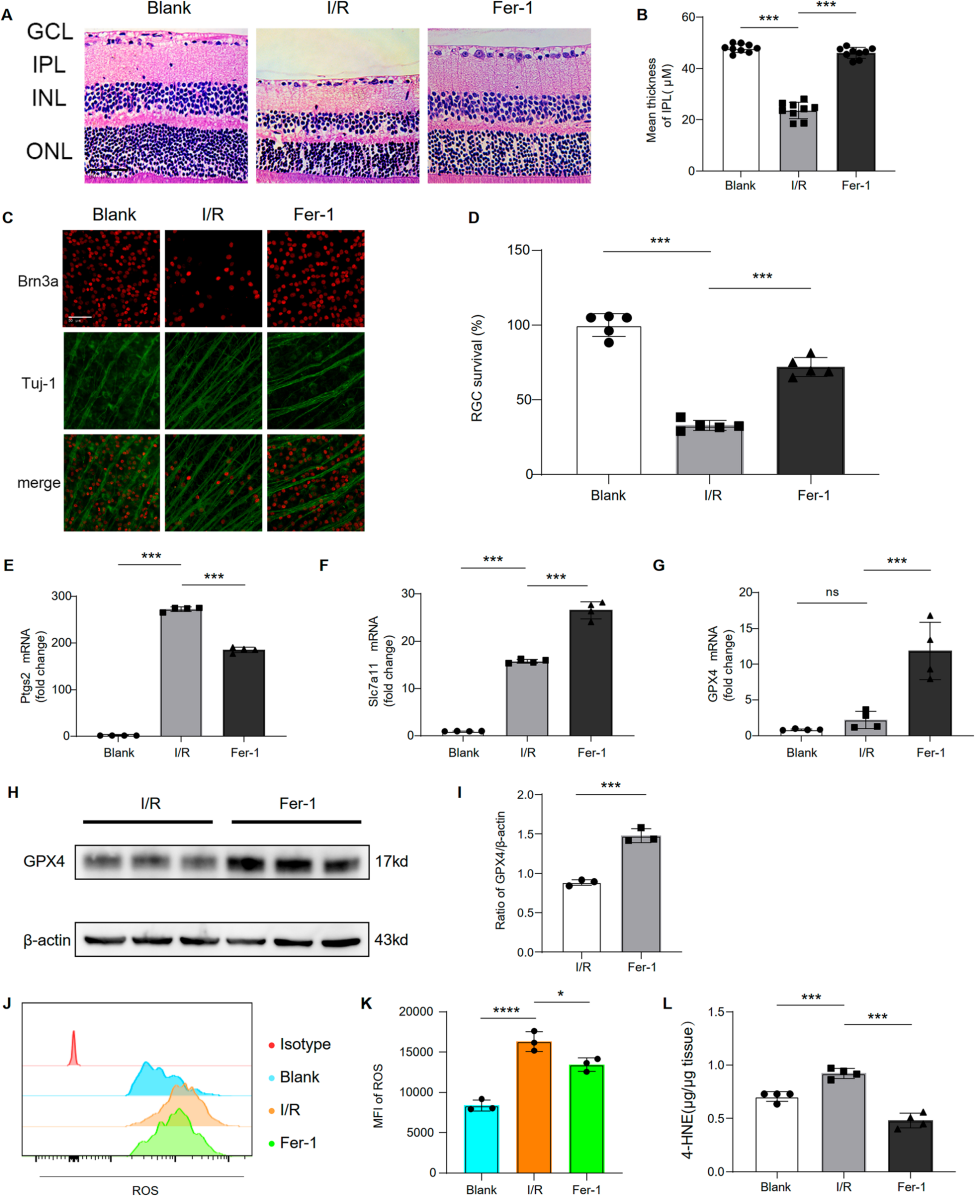
Inhibiting ferroptosis attenuated I/R injury and RGC loss. **A** Representative H&E-stained images among groups. **B** Bar plots showing the mean thickness of IPL among groups (*n* = 9/group). **C** Immunofluorescence image labeling RGCs in retina with Tuj-1 (green) and Brn3a (red), and the merge image of two channels were shown above (scale bar = 50 μm). **D** Bar plots showing the RGC survival rate among groups (*n* = 5/group). The mRNA expression levels of Ptgs2 (**E**), Slc7a11 (**F**), and Gpx4 (**G**) in retina cells were measured with real-time quantitative PCR. Retina lysates were collected and the proteins were subjected to western blot analysis to detect the level of GPX4 (β-actin was used as a control) (**H**, **I**). **J** Flow cytometry histogram showing the expression of ROS in retinal tissue. **K** MFI graph showing the expression of ROS in retinal tissue (*n* = 3/group). **L** Level of 4-HNE in retina tissue of among groups (*n* = 4/group)

Since our scRNA-seq data showed that I/R causes multi-cell ferroptosis, we hypothesized that targeting ferroptosis could mitigate tissue damage caused by I/R. Therefore, we evaluated whether ferroptosis inhibitor (Ferrostatin-1, Fer-1) could reverse I/R-induced tissue injury and RGC death. Retinas exposed to I/R displayed a significantly thinner inner plexiform layer (IPL) 7 days after reperfusion than normal retinas. Damage to the retina, particularly to the IPL, was significantly ameliorated by the Fer-1 treatment (Fig. 6B). RGC apoptosis is another important indicator of functional retinal damage. Immunofluorescence staining of retinal markers confirmed that the RGC number in the I/R mice 7 days after reperfusion was smaller than in the blank mice. Intravitreal Fer-1 injections significantly decreased the severity of retinal damage and the extent of RGC death (Fig. 6C, D).

Next, we studied the neuroprotective mechanism of Fer-1. The disturbed expression of ferroptosis-related genes (Ptgs2, Slc7a11, and Gpx4) was confirmed by real-time PCR. Ptgs2 and Slc7a11 were significantly upregulated after I/R. Ptgs2 was markedly downregulated, and Slc7a11 and Gpx4 upregulated when Fer-1 treatment was included (Fig. 6E–G). The increased expression of Gpx4, induced by Fer-1, was also confirmed by western blot (Fig. 6H, I). Since ferroptosis is a regulated cell death process caused by the unbalanced ROS production and degradation inside the cells. We next explored the effects of Fer-1 on the reactive oxygen species (ROS) and found that the I/R-induced increased ROS levels in the retina were reversed by the Fer-1 treatment (Fig. 6J, K). Furthermore, the Fer-1 treatment reversed the I/R-induced increase in 4-HNE, a ferroptosis metabolite (Fig. 6L). Collectively, treatment with Ferrostatin-1 could reduce I/R-induced retinal injury.

### Ferrostatin-1 inhibited inflammation and cell activation in vivo and in vitro

To investigate whether fer-1 could alleviate the activation of macroglia following retinal I/R, we used GFAP, a specific marker of macroglia. Immunofluorescence results shows the expression of GFAP was gradually increased after retinal I/R and reached a peak at day 7 by the enlarged GFAP-positive cell body and the extending synapses from NFL to ONL. Whereas fer-1 treatment significantly inhibited the upregulation of GFAP expression (Additional file 1: Fig. S3J).

**Fig. 7:**
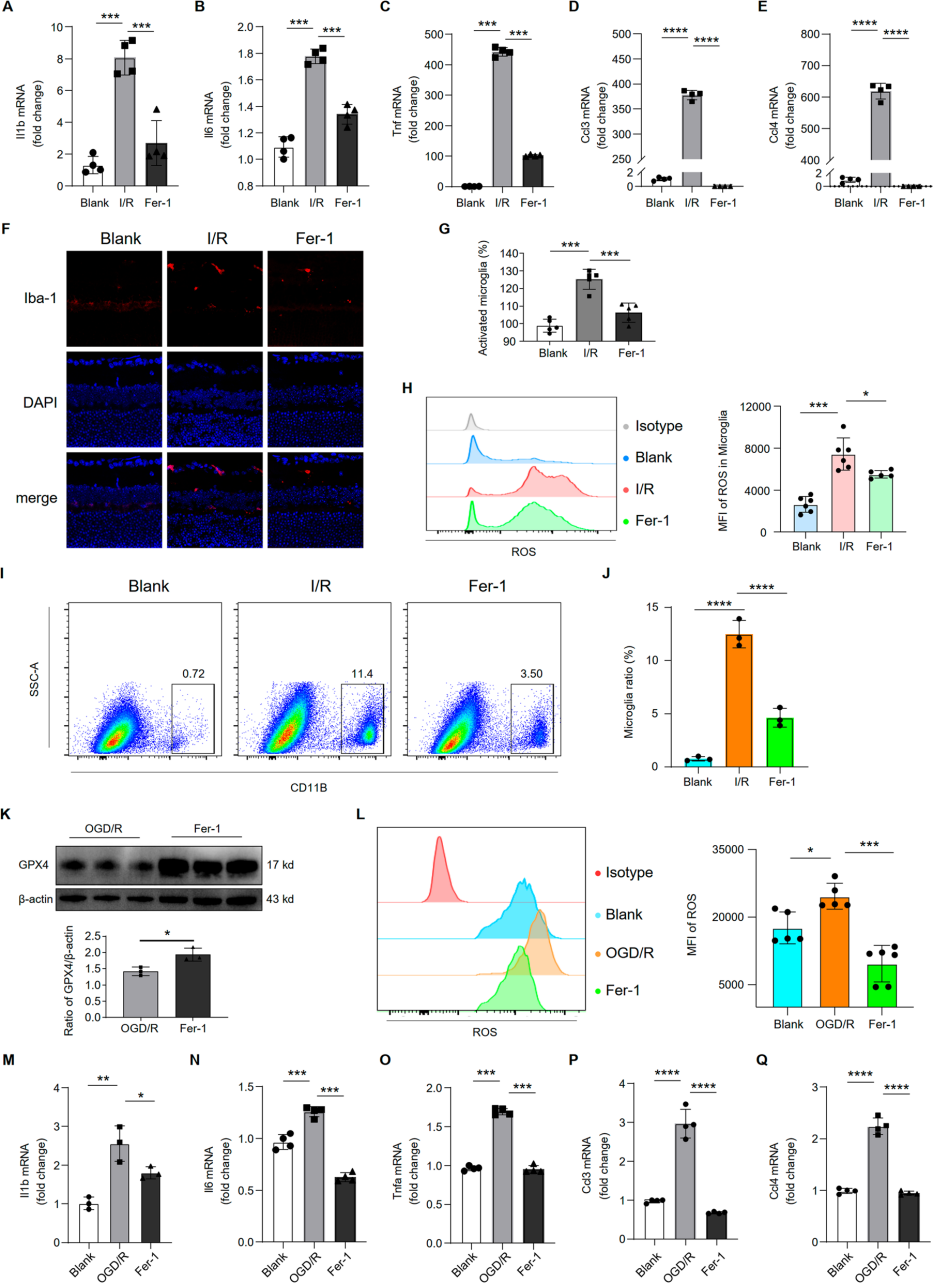
Inhibiting ferroptosis inhibited inflammatory response and cell activation in vivo and vitro. The mRNA expression levels of Il1b (**A**), Il6 (**B**), Tnfa (**C**), Ccl3 (**D**) and Ccl4 (**E**) in retina cells were measured with real-time quantitative PCR. **F** Immunofluorescence image labeling activated microglia with Iba1(red) is shown. Cell nucleuses were stained with DAPI (blue). **G** Statistical analysis of the proportion of IBA1+ cells among groups (*n* = 5/group). **H** Flow cytometry histogram (left) and MFI graph (right) showing the expression of ROS in retinal IBA1+ cells (*n* = 5/group). **I** Flow cytometry histogram showing the expression of CD11b+ cells in retinal tissue. **J** Statistical analysis of the proportion of CD11b+ microglia among groups (*n* = 3/group). **K** Primary retinal microglia lysates were collected and the proteins were subjected to western blot analysis to detect the level of GPX4 (β-actin was used as a control) among groups (*n* = 3/group). **L** Flow cytometry histogram (left) and MFI graph (right) showing the expression of ROS in purified microglia (*n* = 5/group). The mRNA expression levels of Il1b (**M**), Il6 (**N**), Tnfa (**O**), Ccl3 (**P**) and Ccl4 (**Q**) in microglia were measured with real-time quantitative PCR

Previous studies have shown that inflammatory activation of IBA-1 positive cells was the main cause of function loss and RGC death. We have shown that I/R induced upregulation of inflammatory pathways and ferroptosis in IBA-1 positive cells. The cell–cell interactions demonstrated that I/R enhanced the migration and inflammatory activation of myeloid, as shown by the overactivation of the CCL and TNF pathways. We explored Fer-1 effects on inflammation and IBA-1 positive cells activation to further verify its protective effects against I/R damage. Notably, Fer-1 treatment significantly downregulated the mRNA levels of genes involved in IBA-1 positive cells activation and the inflammatory response, including cytokines (Il-1β, Il-6, and TNF-α) and chemokines (CCL3 and CCL4) (Fig. 7A–E). Immunostaining showed that the I/R-induced IBA-1 (a microglial activation marker) positive cells was remarkably attenuated by Fer-1 (Fig. 7F, G). The increase of CD11b+ cells in I/R and their decrease after Fer-1 treatment were also validated by flow cytometry (Fig. 7I, J). Notably, Fer-1 treatment reduced the ROS levels in the retinal CD11b+ cells (Fig. 7H). These results suggested that ferroptosis inhibition by Fer-1 protected against I/R-induced retinal injury by inhibiting IBA-1 positive cells activation and the inflammatory response in vivo.

Next, we tested whether Fer-1 had similar effects on microglia in vitro. We used flow cytometry to sort primary microglia to > 70% purity (Additional file 1: Fig. S4A, B). CCK8 assays assessed Fer-1 cytotoxicity in microglia. We found that Fer-1 showed no substantial cytotoxicity at concentrations ≤ 15 µg/ml (Additional file 1: Fig. S3K). Similarly, we examined the expression of GPX4 in vitro. Western blot showed that Fer-1 treatment could markedly up-regulate the expression of GPX4 in microglia after OGD/R (Fig. 7K). We then tested ROS release in primary microglia in vitro and found its level in the OGD/R group significantly higher than the blank group or following treatment with Fer-1 (1 µg/ml; Fig. 7L). In addition, OGD/R induced an increase in inflammatory genes Il-1β, Il-6, and TNF-α in microglia, which was reversed by treatment with fer-1 (Fig. 7M–O). Meanwhile, Fer-1 inhibited the OGD/R-induced upregulation of CCL3 and CCL4 (Fig. 7P, Q). Collectively, these results suggested that Fer-1 effectively alleviated microglia oxidative stress and inflammatory response in vivo and in vitro.

## Discussion

We applied scRNA-seq analysis and, for the first time, provided single-cell transcriptomics that examined the gene expression in mice with retinal I/R injury. We comprehensively understood the I/R impact on various retinal cell types using cell-type composition, subset-specific gene expression, enriched pathways, and cell–cell communication. The major findings of this study were: (i) the scRNA-seq technology revealed unique gene signatures of retinal cells in mice with retinal I/R injury; (ii) ferroptosis genes’ expression was upregulated in photoreceptor cells, RGC, and glial cells; (iii) ferroptosis occurred in RGC after retinal I/R, alleviated, at least in part, by Fer-1 inhibition of ferroptosis; (iv) the myeloid participated in ferroptosis development after retinal I/R, while Fer-1 attenuated I/R-induced IBA-1 positive cells activation and neuroinflammation.

The vertebrate retina is one of the most well-characterized regions of the central nervous system [30, 31]. It serves as a good model system for studying the neurological impairment mechanisms following I/R. While extensive investigations have well-characterized I/R-related diseases using bioinformatic methods [32–34], the transcriptional features of retinal I/R have not been explored using high-throughput approaches, and a detailed retinal I/R cell atlas was missing before this study. It was very important to reveal the retinal cells’ transcription characteristics to understand the damage caused by I/R. To our knowledge, this was the first study to delineate the retinal cell landscapes of both I/R and blank mice. We identified 12 cell types and described their proportional variations during I/R.

So far, our study has first identified the influx of three immune cell types, myeloid, Neutrophil, and T&DC, dominated the acute inflammatory phase at a single-cell level, accounting for the known inflammatory cascade accumulated by the damage of the blood-eye barrier during I/R [35–37]. These findings suggest that targeted regulation of hyperexpressed genes in the blood-derived immune cells, or inhibition of their recruitment, may be a new approach for protective treatment of retinal ischemia–reperfusion injury, including glaucoma. Although the ratio of retinal Mag decreased, the Mag subsets increase following I/R might be associated with considerable immune cell infiltration and a subsequent decrease in Mag proportion. Furthermore, it has been suggested that RGC, CBC, and the photoreceptors were highly sensitive to I/R [38, 39]. Almost all neuronal cell types were damaged in our study, with ROD seemingly more than CONE. The mouse I/R retinal atlas illustrated aspects of cell-type diversity and function, and revealed transcriptomic and cell-type compositional differences.

Cell death plays a critical role in the response to various stresses and the activation of the immune system. Studies have emphasized the role of deleterious inflammation and oxidative stress in various cell death modes of I/R, including apoptosis, necroptosis, and pyroptosis [40–43]. However, blocking one of these signaling pathways cannot completely ameliorate inflammation, suggesting the mutual involvement of several cell death pathways, as indicated by GO analysis of various cell types in the I/R retinas. Apoptosis, the most well-characterized PCD to date, is immunologically silent (“clean”) and its activation does not promote a significant inflammatory or autoimmune response [44, 45]. The non-apoptotic PCDs, including necroptosis, pyroptosis, and ferroptosis, are a much “dirtier”. The most well-characterized inflammation associated cell death models defined primarily by acute injury, including those induced by I/R, pathogenic infection, and neurodegenerative and neuroinflammatory diseases. These pathologies have a significant combination of cell death components, resulting in a severe inflammatory state. Furthermore, considering the role of microenvironmental molecular changes in RGC damage, we showed through ligand–receptor interaction map that the SEMA3 pathway was downregulated in RGC after I/R. The SEMA3 pathway is known for its role in axon guidance, facilitating retinal axon crossing in the chiasm [26], possibly accounting for the difficulty in nerve regeneration after I/R damage. In summary, retinal I/R-induced nerve injury involves various cell death pathways.

Of importance, ferroptosis overexpression in almost all cell types can enhance our understanding of retinal I/R. Unlike the other classical non-apoptotic PCD, ferroptosis is a unique PCD characterized by iron-dependent accumulation of lipid hydroperoxides to lethal levels [46–48]. Iron homeostasis is tightly regulated as its deficiency and overload could lead to various pathological conditions [49, 50]. Excessive iron release can induce intraocular peroxidation of unsaturated phospholipids and retinal inflammation [51, 52]. Iron intake might increase the risk of glaucoma [53]; however, very little research has been done in this area. Indeed, iron uptake and transport were found in our GO pathway analysis, suggesting iron imbalance in I/R retinas. Emerging evidence indicated that ferritin light chain 1(Ftl1) and ferritin heavy chain 1 (Fth1) were involved in iron storage, entry, and homeostasis [54–58]. Our DEG analysis found that these were upregulated in most of cell subsets. Ferroptosis and iron homeostasis imbalance have been implicated in several neuroinflammatory diseases, including ischemic stroke, subarachnoid hemorrhage, Alzheimer’s disease, and Parkinson disease [59, 60]. Our cell atlas and bioinformatic methods revealed considerable enrichment of ferroptosis response gene networks (e.g., Slc7a11, Gpx4 and Ptgs2) in the I/R retinas in vivo and in vitro. Ferroptosis onset involves an intracellular iron overload and upregulation lipid peroxides. Ptgs2, Slc7a11 and Gpx4 are widely accepted ferroptosis biomarkers (57, 63). Networks revolving around glutathione peroxidase 4 (GPX4) regulate ferroptosis, among which the system Xc^−^–glutathione–GPX4 axis is most well-known one [55, 61]. Slc7a11, a key component of the cystine/glutamate antiporter system Xc^−^, transfers cystine to glutathione biosynthesis and regulates cellular lipid peroxidation [54–58, 62]. The expression or activity of Gpx4 restrain lipid peroxides at the expense of GSH [63]. In models of CNS injury, microglial activation has been shown to induce iron overload and subsequently trigger neuronal ferroptosis [64]. We showed that ferroptosis inhibitors reduced inflammatory cytokines, partially prevented RGC death, and mitigated the activation of IBA-1 positive cells, the executor of PCD and mediator of immune storm. These findings provide compelling evidence that ferroptosis is a promising therapeutic target for immune programming. In a newly published study, Qin and colleagues investigated the interactions among different modes of cell death (apoptosis, necroptosis, and ferroptosis) in RGC loss during retinal I/R injury [65], which is consistent with our findings. In addition to RGC, we discovered that almost all retinal cells, such as CONE, ROD, Mag and myeloid underwent ferroptosis with retinal I/R injury as well. Importantly, ferroptosis in myeloid took a non-negligible part in retinal I/R injury by recruiting inflammatory factors and amplifying inflammatory cascades. Given the strong pro-inflammatory effects of ferroptosis [62], its regulation could help control the inflammatory and immune responses, crucial knowledge when designing optimized therapies for retinal I/R-induced neuroinflammation. Targeted inhibitors could alleviate ferroptosis-mediated neuronal damage and reduce neuroinflammation by IBA-1 positive cells activation.

Post-I/R inflammation is a complex process involving diverse signaling pathways and various cell types. Cell death has been reported to trigger neuroinflammation following plasma membrane bursting and subsequent promotion of pro-inflammatory responses from the immune system [66–68]. Microglia, the immunocompetent cells of the CNS and the first responders to neuronal injury and death, become reactive upon retinal I/R and produce various cytokines, including TNF-α, IL-6, and IL-1β [69–71]. We and others have investigated the mechanisms underlying myeloid cell-mediated initiation and resolution [11, 72] of inflammation. Our findings demonstrate that myeloid are the main effector cells to induce cell mediator release and inflammatory infiltration. Upregulated inflammatory cytokines (IL-1, IL-6, and TNF-α) and pathways (CCL, TNF, and myeloid leukocyte activation) are involved in inflammatory spreading. In turn, inhibited ferroptosis in myeloid, combined with a reduction of cell death, resulted in the inhibition of myeloid activation. Therefore, targeting retinal I/R injury induced ferroptosis can “kill two birds with one stone” by inhibiting cell death-mediated damage and reducing inflammatory activation. Since ferroptosis was the only PCD mode overexpressed in almost all retinal cells, we showed here for the first time that Fer-1 reduced inflammatory cytokines and IBA-1 positive cells activation, and enhanced RGC survival. Furthermore, similar to the synchronized responses of macroglia and IBA-1 positive cells to neuron injury, their bi-directional interactions are critical for building and amplifying neuroinflammation and dictating neurologically compromised outcomes [73]. Müller cells, the principal retinal macroglia, undergo reactive gliosis after acute injury or chronic neuronal stress, which exerts dual functions [74]. GO analysis of Müller cells indicated the induction of a pro-inflammatory response through the upregulation of the IFNG and MAPK pathways. In addition, we discovered an interesting phenomenon, in which inflammatory pathways were upregulated in non-immune cell subsets, including RGC and photoreceptor cells. Traditionally, photoreceptor cells were assumed highly specialized, terminally differentiated neurons that detect photons and transmit light information to bipolar cells in the retina [75]. However, their structural and metabolic requirements render them susceptible to many acquired and genetic injury sources. GO analysis of photoreceptor subsets showed that CONE-SC3 and ROD-SC2 were specifically involved in inflammatory responses (MAPK and IFNG signaling pathways). These novel evidences suggest that ferroptosis may be a new target for retinal I/R injury diseases, including retinal vessel occlusion, diabetic retinopathy and glaucoma. However, the exact mechanism of ferroptosis has not been elucidated yet. In addition, most of the existing ferroptosis inhibitors are non-specific, which could also lead to the limitation of clinical drug research. Therefore, targeting I/R-induced neuroinflammation by blocking ferroptosis might be an attractive strategy to treat retinal I/R injury.

## Conclusions

In conclusion, our study delineated a retinal cell atlas of mice with I/R and explored the cell types involved in neuroinflammation mediation. We demonstrated the critical role of ferroptosis in I/R, and the ability of using ferroptosis inhibitor to alleviate RGC death and neuroinflammation. Importantly, this was the first study to reveal the role of ferroptosis in regulating neuroinflammation and RGC death in retinal I/R injury. Ferroptosis unique signaling pathways might help identify potential treatment targets for neuroinflammatory diseases, especially glaucoma, and thus protect neuronal cells.

## Supplementary Information

**Additional file 1: Fig. S1.** I/R induced altered heterogeneity in retinal cell constitution. **A** t-SNE distribution showing a group of myeloid cells with high expression of Itgam and Lyz2, as well as cell subsets with high expression of C1qa and Ly6c2. **B** Pie plot showing the relative ratio of 12 clusters between Blank (left) and I/R (right) groups. **C** Violin plots showing the proportion of 4 clusters in retinal cells between Blank and I/R groups (*n* = 3/group). *P* value were calculated using a Wilcoxon rank-sum test. **D** t-SNE distribution shows strategies for CONE clustering and subclassification from retinal cells. **E** t-SNE distribution shows strategies for ROD clustering and subclassification from retinal cells. **F** Violin plots showing the proportion of 3 ROD-subclusters between Blank and I/R groups (*n* = 3/group). *P* value were calculated using a Wilcoxon rank-sum test. **G** Inflammation-related GO and pathway enrichment analysis of the CONE-SCs clustering DEGs. *P* value was derived by a hypergeometric test. **H** Inflammation-related GO and pathway enrichment analysis of the CONE-SCs clustering DEGs. *P* value was derived by a hypergeometric test. **Fig. S2.** I/R induced an expansion of macroglia subclusters with ferroptosis. **A** t-SNE distribution showing 7 subclusters in macroglia. **B** t-SNE distribution showing groups (Blank and I/R) in macroglia. **C** Bar plots showing cell abundances across Mag-SCs (*n* = 7) for the Blank and I/R groups. **D** Violin plots showing the proportion of Mag-SC0 in CONE between Blank and I/R groups (*n* = 3/group). *P* value were calculated using a Wilcoxon rank-sum test. **E** Violin plots showing the proportion of 3 Mag-SCs in CONE between Blank and I/R groups (*n* = 3/group). *P* value were calculated using a Wilcoxon rank-sum test. **F** A heat map representing the scaled expression values of the top 10 genes defining each Mag-SCs. **G** Violin plots showing the expression of gliocyte-related genes in Mag-SCs. **H** GO and pathway enrichment analysis of the Mag-SCs clustering DEGs. P value was derived by a hypergeometric test. **Fig. S3.** I/R injury induced an expansion of Myeloid subclusters with ferroptosis and inflammation activation. **A** Volcano plot showing DEGs that are I/R-upregulated (red) or downregulated (blue) in Myeloid. *P* value were calculated using a Wilcoxon rank-sum test. **B** t-SNE distribution shows strategies for Myeloid clustering and subclassification from retinal cells. **C** Violin plots showing the inflammatory response score (left) and ferroptosis scores (right) between Blank and I/R groups among microglia cluster. **D** Violin plots showing the expression of genes related to IGF signaling between Blank and I/R groups. **E** Violin plots showing the expression of genes related to NT signaling between Blank and I/R groups. **F** Heatmap shows the role and importance of each cluster in SEMA3 signaling network (up). Bar plots showing the relative contribution of each ligand–receptor pair in SEMA3 signaling pathway (down). **G** Circle plot showing the inferred TNF signaling networks between Blank and I/R groups. **H** Bar plots showing the relative contribution of each ligand–receptor pair in CCL signaling pathway. **I** Violin plots showing the expression of genes related to CCL signaling between Blank and I/R groups. **J** Immunofluorescence image labeling activated macroglia with GFAP(red) is shown. Cell nucleuses were stained with DAPI (blue) (scale bar = 50 μm). **K** The cell viability showing the cytotoxicity of Fer-1 on primary microglia. **Fig. S4.** Sorting strategy for CD11B+ microglia in vitro. **A** Flow cytometry histogram showing the sorting strategy for CD11B+ microglia. **B** Percentage showing the purification of primary microglia.

**Additional file 2: Table S1.** Primary antibodies and dilutions. **Table S2.** Primer sequences used for real-time PCR. **Table S3.** Numbers of each cell cluster in all single-cell RNA sequencing samples. **Table S4.** Raw data of clustering analysis for single-cell RNA sequencing.

## Data Availability

The data are available from the corresponding author on reasonable request.

## Abbreviations

I/R: Ischemia–reperfusion
TLR4: Toll-like receptor 4
scRNA-seq: Single-cell RNA sequencing
Fer-1: Ferrostatin-1
tSNE: T-distributed stochastic neighbor embedding
CBC: Cone bipolar cell
RBC: Rod bipolar cell
AC: Amacrine cell
Mag: Macroglia
HC: Horizontal cell
RGC: Retina ganglion cell
VEC: Vascular endothelial cell
Myeloid: Monocyte, macrophage and microglia
T&DC: T cell and dendritic cells
DEG: Differentially expressed gene
KEGG: Kyoto Encyclopedia of Genes and Genomes
SCs: Subclusters
GO: Gene ontology
IPL: Inner plexiform layer
ROS: Reactive oxygen species
PCD: Programmed cell death
GSH: Glutathione
CNS: Central nervous system
