# Supplementary material for: Single-cell RNA sequencing reveals a landscape and targeted treatment of ferroptosis in retinal ischemia/reperfusion injury
Source: J Neuroinflammation. 2022 Oct 26;19:261. doi: 10.1186/s12974-022-02621-9 (PMC9597965; doi:10.1186/s12974-022-02621-9)
Supplement: Supplementary file 1 — Additional file 1: Fig. S1. I/R induced altered heterogeneity in retinal cell constitution. A t-SNE distribution showing a group of myeloid cells with high expression of Itgam and Lyz2, as well as cell subsets with high expression of C1qa and Ly6c2. B Pie plot showing the relative ratio of 12 clusters between Blank (left) and I/R (right) groups. C Violin plots showing the proportion of 4 clusters in retinal cells between Blank and I/R groups (n = 3/group). P value were calculated using a Wilcoxon rank-sum test. D t-SNE distribution shows strategies for CONE clustering and subclassification from retinal cells. E t-SNE distribution shows strategies for ROD clustering and subclassification from retinal cells. F Violin plots showing the proportion of 3 ROD-subclusters between Blank and I/R groups (n = 3/group). P value were calculated using a Wilcoxon rank-sum test. G Inflammation-related GO and pathway enrichment analysis of the CONE-SCs clustering DEGs. P value was derived by a hypergeometric test. H Inflammation-related GO and pathway enrichment analysis of the CONE-SCs clustering DEGs. P value was derived by a hypergeometric test. Fig. S2. I/R induced an expansion of macroglia subclusters with ferroptosis. A t-SNE distribution showing 7 subclusters in macroglia. B t-SNE distribution showing groups (Blank and I/R) in macroglia. C Bar plots showing cell abundances across Mag-SCs (n = 7) for the Blank and I/R groups. D Violin plots showing the proportion of Mag-SC0 in CONE between Blank and I/R groups (n = 3/group). P value were calculated using a Wilcoxon rank-sum test. E Violin plots showing the proportion of 3 Mag-SCs in CONE between Blank and I/R groups (n = 3/group). P value were calculated using a Wilcoxon rank-sum test. F A heat map representing the scaled expression values of the top 10 genes defining each Mag-SCs. G Violin plots showing the expression of gliocyte-related genes in Mag-SCs. H GO and pathway enrichment analysis of the Mag-SCs clustering DEGs. P v [file 12974_2022_2621_MOESM1_ESM.pdf]

Figure S1

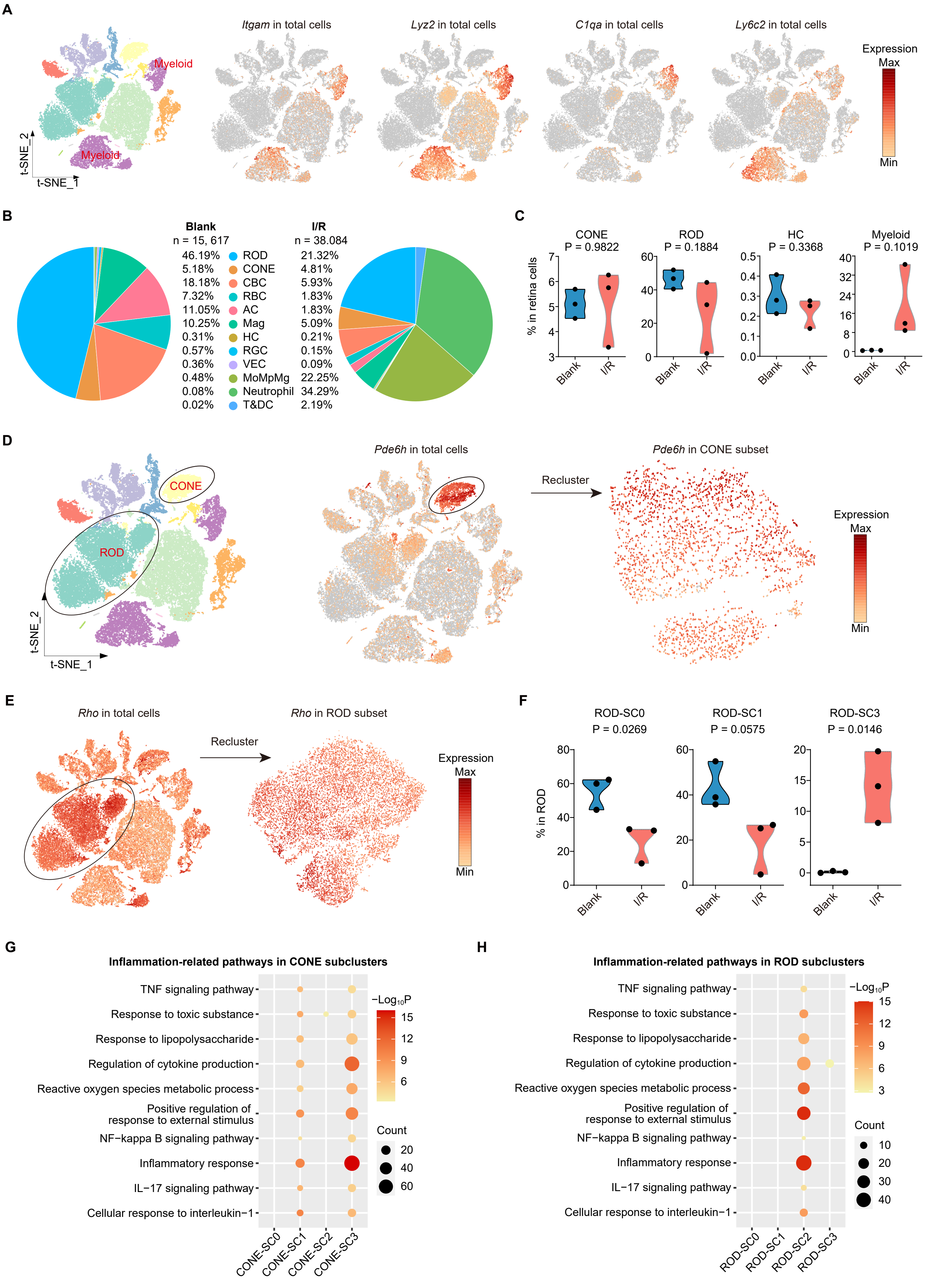

A
Myeloid
Myeloid
t-SNE_2
t-SNE_1
Itgam in total cells
Lyz2 in total cells
C1qa in total cells
Ly6c2 in total cells
Expression
Max
Min
B
Blank
n = 15, 617
I/R
n = 38.084
46.19% ROD 21.32%
5.18% CONE 4.81%
18.18% CBC 5.93%
7.32% RBC 1.83%
11.05% AC 1.83%
10.25% Mag 5.09%
0.31% HC 0.21%
0.57% RGC 0.15%
0.36% VEC 0.09%
0.48% MoMpMg 22.25%
0.08% Neutrophil 34.29%
0.02% T&DC 2.19%
C
CONE
P = 0.9822
ROD
P = 0.1884
HC
P = 0.3368
Myeloid
P = 0.1019
% in retina cells
Blank
I/R
D
CONE
ROD
t-SNE_2
t-SNE_1
Pde6h in total cells
Recluster
Pde6h in CONE subset
Expression
Max
Min
E
Rho in total cells
Recluster
Rho in ROD subset
Expression
Max
Min
F
ROD-SC0
P = 0.0269
ROD-SC1
P = 0.0575
ROD-SC3
P = 0.0146
% in ROD
Blank
I/R
G
Inflammation-related pathways in CONE subclusters
TNF signaling pathway
Response to toxic substance
Response to lipopolysaccharide
Regulation of cytokine production
Reactive oxygen species metabolic process
Positive regulation of response to external stimulus
NF−kappa B signaling pathway
Inflammatory response
IL−17 signaling pathway
Cellular response to interleukin−1
CONE-SC0
CONE-SC1
CONE-SC2
CONE-SC3
−Log10P
15
12
9
6
Count
20
40
60
H
Inflammation-related pathways in ROD subclusters
TNF signaling pathway
Response to toxic substance
Response to lipopolysaccharide
Regulation of cytokine production
Reactive oxygen species metabolic process
Positive regulation of response to external stimulus
NF−kappa B signaling pathway
Inflammatory response
IL−17 signaling pathway
Cellular response to interleukin−1
ROD-SC0
ROD-SC1
ROD-SC2
ROD-SC3
−Log10P
15
12
9
6
3
Count
10
20
30
40

Figure S2

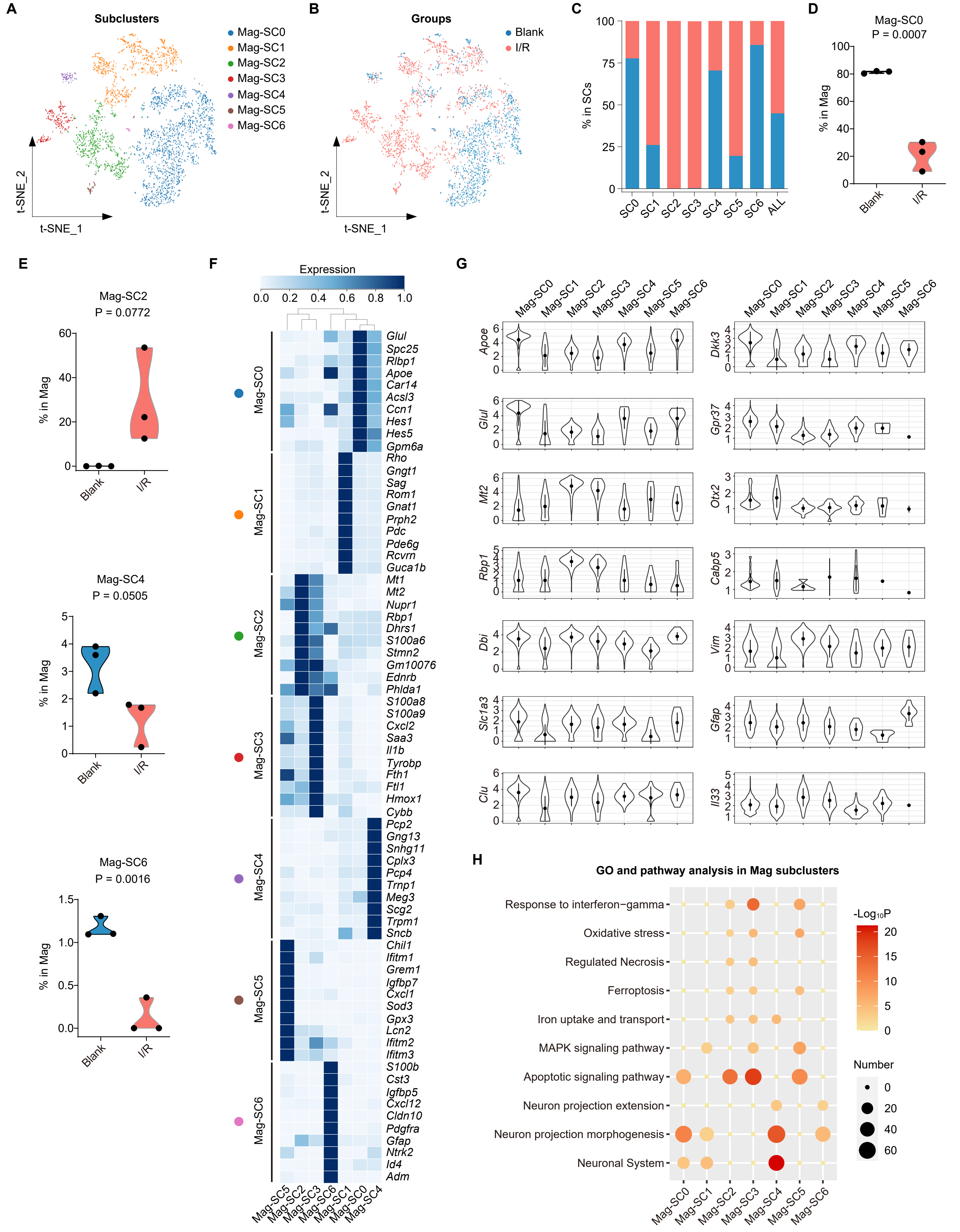

A
Subclusters
Mag-SC0
Mag-SC1
Mag-SC2
Mag-SC3
Mag-SC4
Mag-SC5
Mag-SC6
t-SNE_1
t-SNE_2
B
Groups
Blank
I/R
C
% in SCs
SC0 SC1 SC2 SC3 SC4 SC5 SC6 ALL
D
Mag-SC0
P = 0.0007
% in Mag
E
Mag-SC2
P = 0.0772
Mag-SC4
P = 0.0505
Mag-SC6
P = 0.0016
F
Expression
Glul Spc25 Rlbp1 Apoe Car14 Acsl3 Ccn1 Hes1 Hes5 Gpm6a
Rho Gngt1 Sag Rom1 Gnat1 Prph2 Pdc Pde6g Rcvrn Guca1b
Mt1 Mt2 Nupr1 Rbp1 Dhrs1 S100a6 Stmn2 Gm10076 Ednrb Phlda1
S100a8 S100a9 Cxcl2 Saa3 Il1b Tyrobp Fth1 Ftl1 Hmox1 Cybb
Pcp2 Gng13 Snhg11 Cplx3 Pcp4 Trnp1 Meg3 Scg2 Trpm1 Sncb
Chil1 Ifitm1 Grem1 Igfbp7 Cxcl1 Sod3 Gpx3 Lcn2 Ifitm2 Ifitm3
S100b Cst3 Igfbp5 Cxcl12 Cldn10 Pdgfra Gfap Ntrk2 Id4 Adm
G
Apoe Glul Mt2 Rbp1 Dbi Slc1a3 Clu
Dkk3 Gpr37 Otx2 Cabp5 Vim Gfap Il33
H
GO and pathway analysis in Mag subclusters
Response to interferon−gamma
Oxidative stress
Regulated Necrosis
Ferroptosis
Iron uptake and transport
MAPK signaling pathway
Apoptotic signaling pathway
Neuron projection extension
Neuron projection morphogenesis
Neuronal System
-Log10P
Number

Figure S3

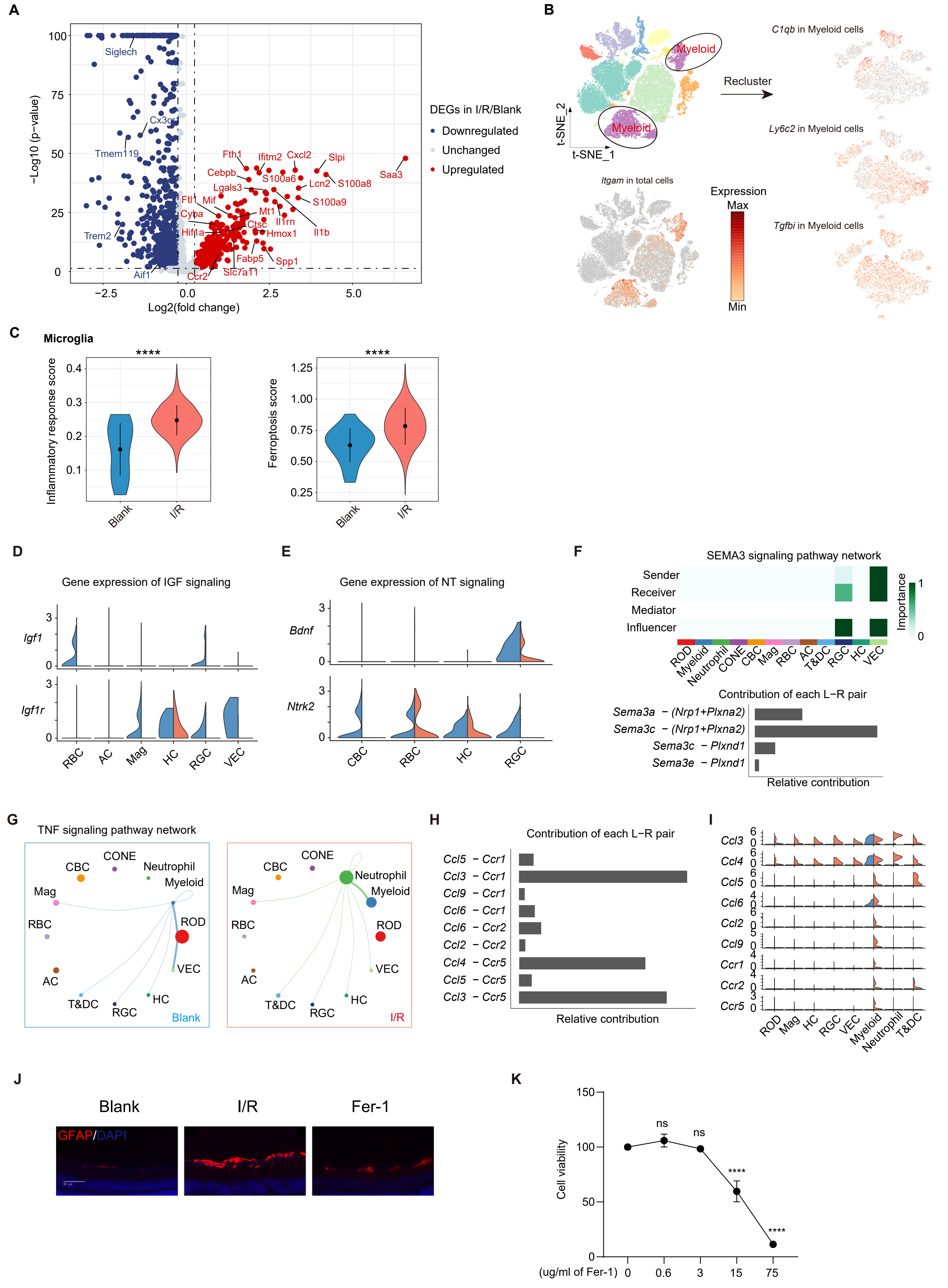

A
DEGs in I/R/Blank
Downregulated
Unchanged
Upregulated
−Log10 (p−value)
Log2(fold change)
Siglech
Cx3cr1
Tmem119
Trem2
Aif1
Fth1
Ifitm2
Cxcl2
Slpi
Saa3
Cebpb
S100a6
Lcn2
S100a8
Lgals3
S100a9
Ftl1
Mif
Cyba
Mt1
Ctsc
Il1rn
Hif1a
Hmox1
Il1b
Fabp5
Spp1
Ccr2
Slc7a11
B
Myeloid
t-SNE_2
t-SNE_1
Recluster
C1qb in Myeloid cells
Ly6c2 in Myeloid cells
Tgfbi in Myeloid cells
Itgam in total cells
Expression
Max
Min
C
Microglia
Inflammatory response score
Ferroptosis score
Blank
I/R
****
D
Gene expression of IGF signaling
Igf1
Igf1r
RBC AC Mag HC RGC VEC
E
Gene expression of NT signaling
Bdnf
Ntrk2
CBC RBC HC RGC
F
SEMA3 signaling pathway network
Sender
Receiver
Mediator
Influencer
Importance
ROD Myeloid Neutrophil CONE CBC Mag RBC AC T&DC RGC HC VEC
Contribution of each L−R pair
Sema3a − (Nrp1+Plxna2)
Sema3c − (Nrp1+Plxna2)
Sema3c − Plxnd1
Sema3e − Plxnd1
Relative contribution
G
TNF signaling pathway network
CONE
CBC
Neutrophil
Myeloid
Mag
RBC
ROD
VEC
AC
T&DC
RGC
HC
Blank
I/R
H
Contribution of each L−R pair
Ccl5 − Ccr1
Ccl3 − Ccr1
Ccl9 − Ccr1
Ccl6 − Ccr1
Ccl6 − Ccr2
Ccl2 − Ccr2
Ccl4 − Ccr5
Ccl5 − Ccr5
Ccl3 − Ccr5
Relative contribution
I
Ccl3
Ccl4
Ccl5
Ccl6
Ccl2
Ccl9
Ccr1
Ccr2
Ccr5
ROD Mag HC RGC VEC Myeloid Neutrophil T&DC
J
Blank
I/R
Fer-1
GFAP/DAPI
K
Cell viability
(ug/ml of Fer-1)
0
0.6
3
15
75
ns
****

Figure S4

A

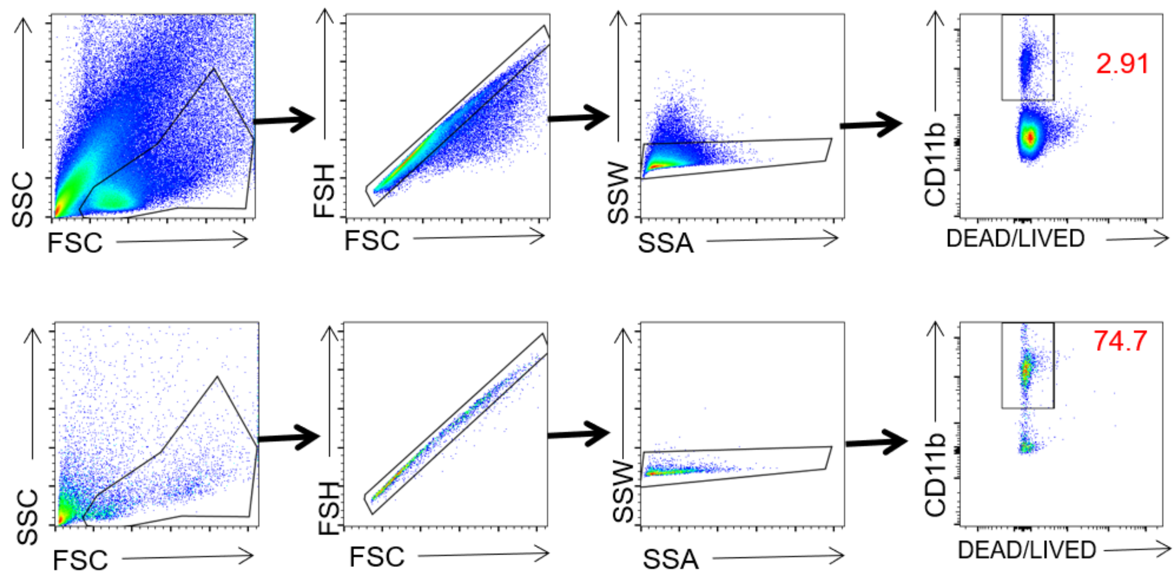

SSC
FSC
FSH
FSC
SSW
SSA
CD11b
DEAD/LIVED
2.91
SSC
FSC
FSH
FSC
SSW
SSA
CD11b
DEAD/LIVED
74.7

B

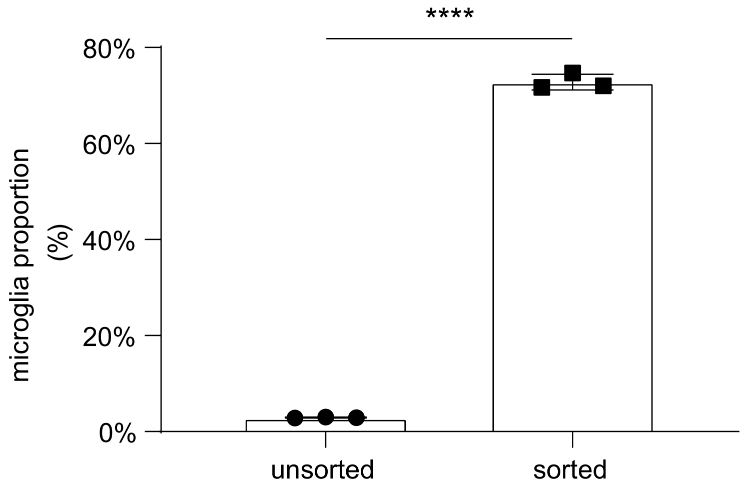

****
80%
60%
40%
20%
0%
microglia proportion (%)
unsorted
sorted
